# Supplementary material for: MRLocus: Identifying causal genes mediating a trait through Bayesian estimation of allelic heterogeneity
Source: PLoS Genet. 2021 Apr 19;17(4):e1009455. doi: 10.1371/journal.pgen.1009455 (PMC8084342; doi:10.1371/journal.pgen.1009455)
Supplement: S1 Text — (PDF) [file pgen.1009455.s002.pdf]

S1 Text: Supplementary Tables and Figures

Anqi Zhu†, Nana Matoba†, Emma P. Wilson, Amanda L. Tapia,
Yun Li, Joseph G. Ibrahim, Jason L. Stein, Michael I. Love

March 10, 2021

eQTL Tissue	Resource
Artery Tibial (GTEx v8)	`https://console.cloud.google.com/storage/browser/gtex-resources`
Blood (eQTLGen 2019-12-23 release)	`https://www.eqtlgen.org/cis-eqtls.html`
Liver	`https://www.nature.com/articles/s41598-018-24219-z`

GWAS Phenotype	Resource
CAD (CARDIoGRAMplusC4D)	`http://www.cardiogramplusc4d.org/media/cardiogramplusc4d-consortium/data-downloads/cad.additive.Oct2015.pub.zip`
HDL (UKBB)	`https://www.dropbox.com/s/65jisgxwbbdrkaw/30760_irnt.gwas.imputed_v3.both_sexes.tsv.bgz`
LDL (UKBB)	`https://www.dropbox.com/s/4rnjzczwjgs5pgl/30780_irnt.gwas.imputed_v3.both_sexes.tsv.bgz`

Table A. Links to URLs for eQTL and GWAS summary data employed in MRLocus real data evaluation.

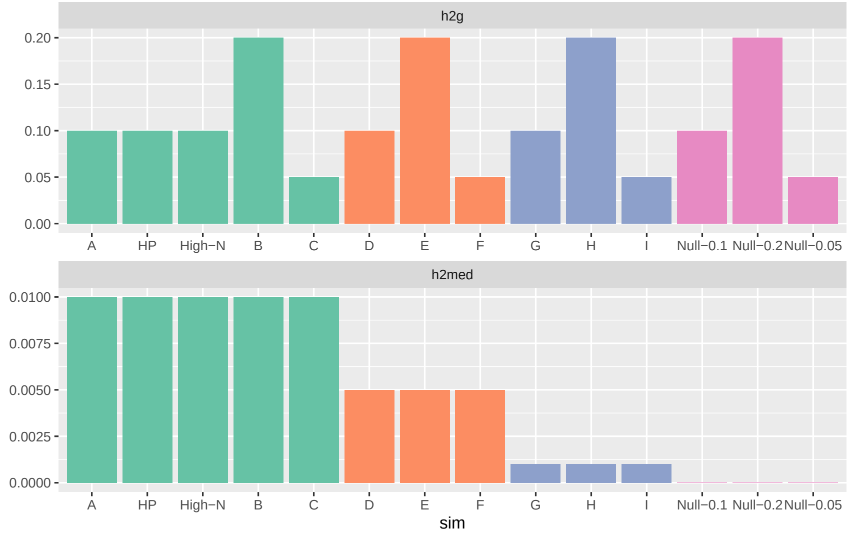

Fig A. Diagram of the 14 types of `twas_sim` simulations performed, varying expression heritability h^2g (top row) and expression mediated heritability h^2med (bottom row). Results for simulation A are presented in Figure 2 in the main text, while results for the remaining 13 simulation settings follow in Supplementary Figures.

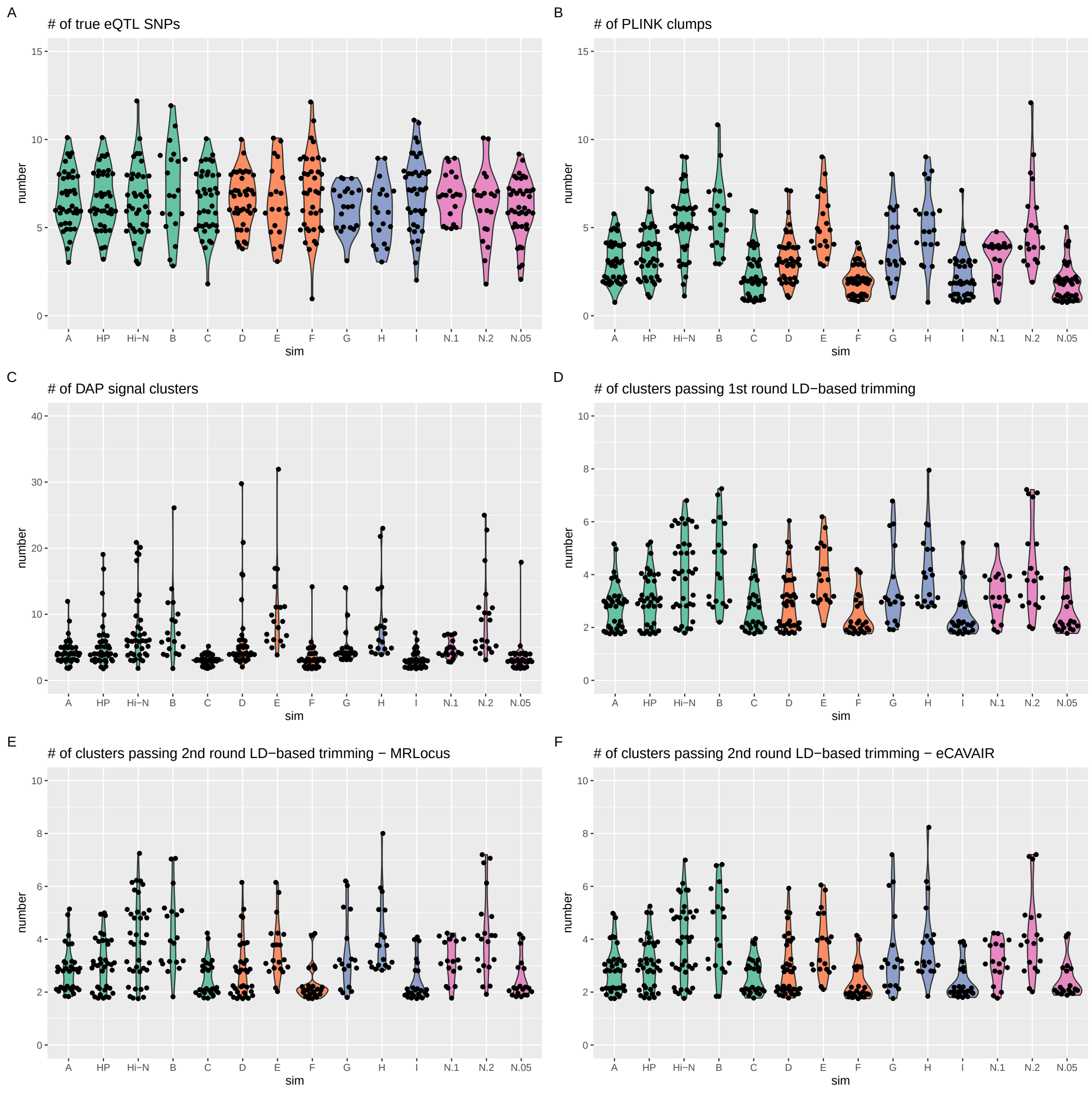

Fig B. Number of (A) true causal eQTL SNPs, (B) PLINK clumps, (C) DAP signal clusters per simulation, and number of clusters passing (D) 1st round LD trimming, (E) 2nd round LD trimming for MRLocus colocalization, and (F) 2nd round LD trimming for eCAVIAR colocalization.

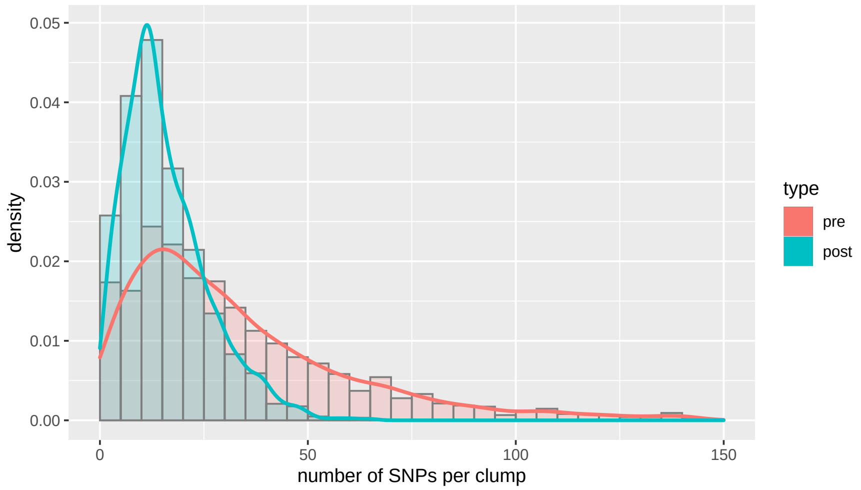

Fig C. SNPs per clump in the 240 simulations, before and after collapsing with MRLocus `collapseHighCorSNPs` function. The mean number of SNPs per clump was 19.9 and 8.8, and the median number of SNPs per clump was 15 and 8 (before and after, respectively).

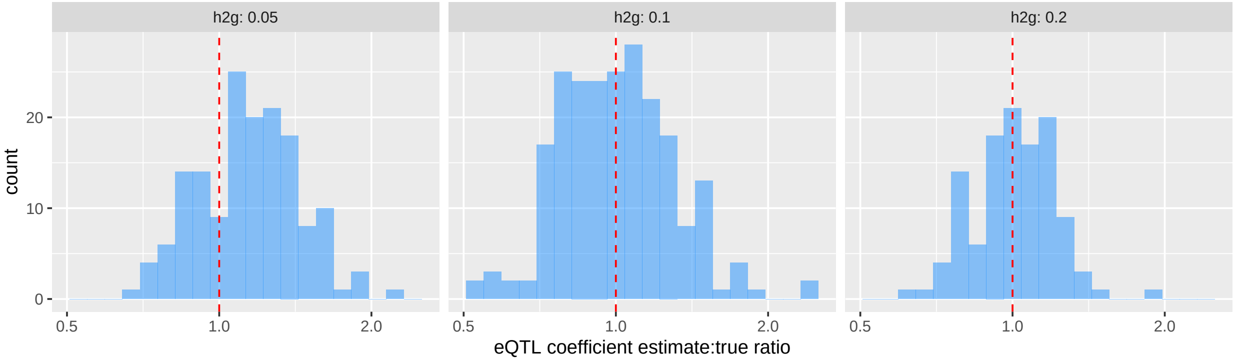

Fig D. Ratio of estimated eQTL coefficient over true value across simulations, grouped by h^2g parameter. Histograms represent the ratio for all causal eSNPs with un-adjusted eQTL p-value less than the clumping threshold (0.001). The median and interquartile range (IQR) of the distributions (on the original ratio scale) are (1.15, 0.37), (1.00, 0.35), and (1.01, 0.25), for the 5%, 10%, and 20% h^2g settings, respectively.

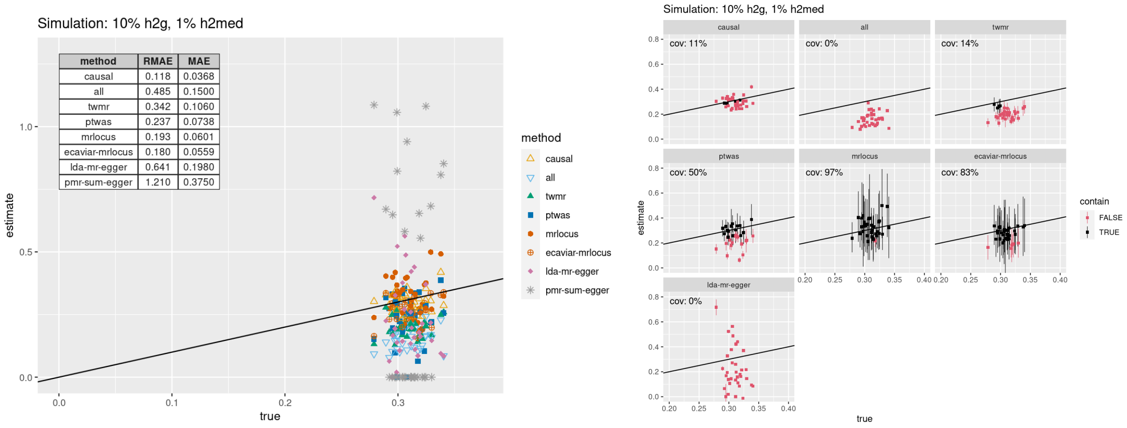

Fig E. Accuracy and interval coverage of gene-to-trait effect estimation in simulation A, including LDA-MR-Egger and PMR-Summary-Egger in comparisons. As PMR-Summary-Egger does not provide a standard error for the causal effect, interval coverage was only computed for LDA-MR-Egger.

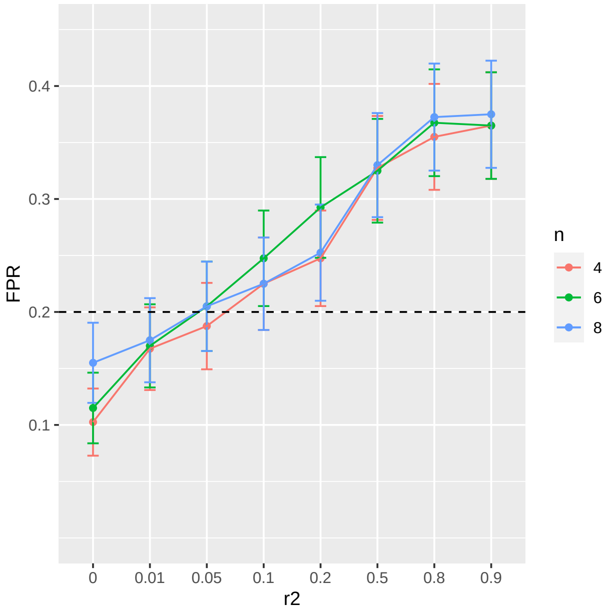

Fig F. Assessment of the effect of correlated instruments on the false positive rate of MRLocus slope estimation. Increasing the r^2 of adjacent signal clusters raised the rate of 80% credible intervals not covering the true value of the slope $\alpha = 0$. The number of signal clusters was varied among $\{4, 6, 8\}$. Bars represent 95% binomial-based confidence intervals, and 400 simulation iterations were performed for each combination of r^2 and the number of signal clusters.

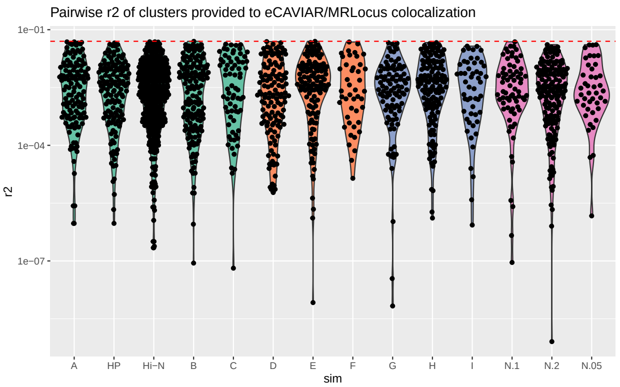

Fig G. Pairwise r^2 across clusters for the instruments provided to MRLocus slope fitting, across all iterations and all simulated datasets. Clusters were trimmed such that $r^2 < 0.05$.

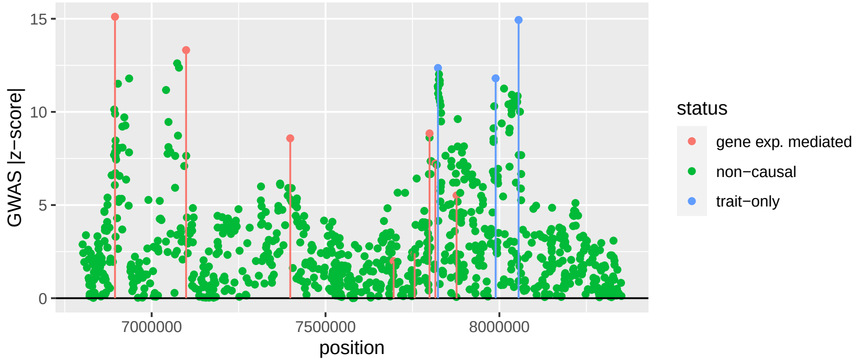

Fig H. Example of one of the iterations of the simulation of partial mediation with horizontal pleiotropy (HP), where three large trait-only association signals are added to a simulation with $h^2g = 10\%$ and $h^2med = 1\%$. Absolute Z-scores for the GWAS population are calculated from estimated coefficients and their standard errors, and colored by the true status in the simulation.

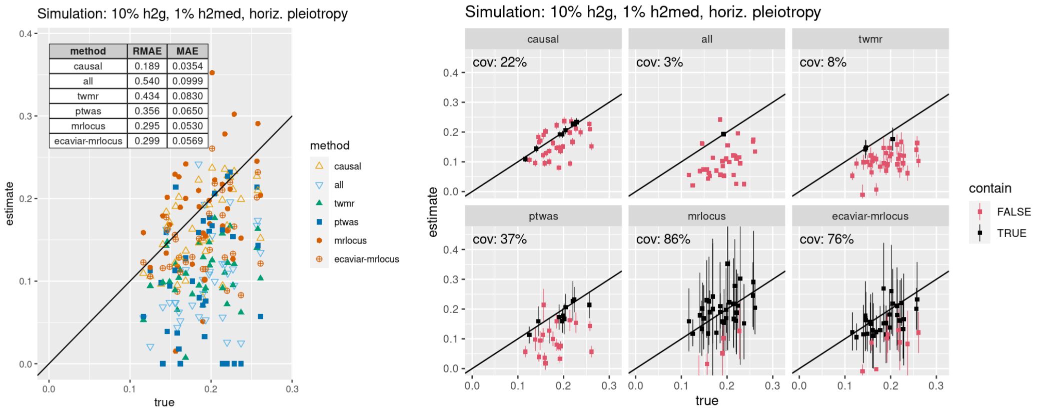

Fig I. Accuracy and interval coverage of TWMR, PTWAS, MRLocus, and eCAVIAR-MRLocus on the simulation of partial mediation with horizontal pleiotropy (as in the example region in Supplementary Figure H).

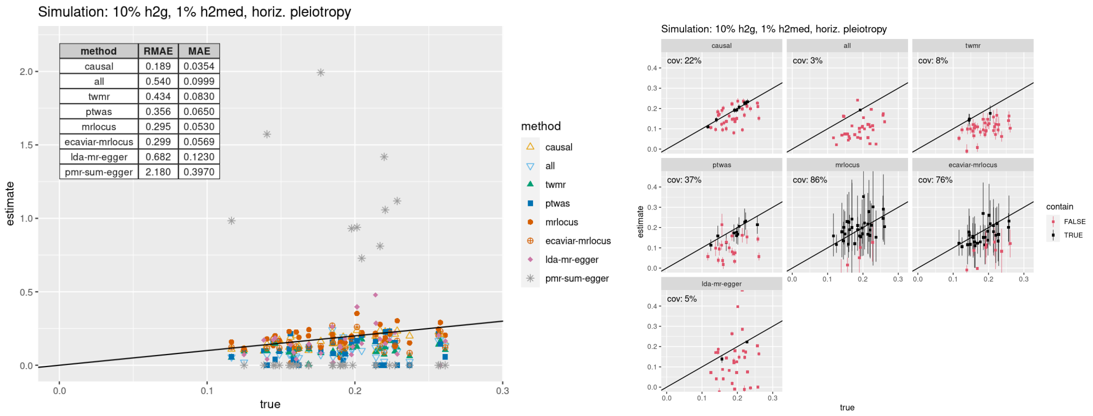

method	RMAE	MAE
causal	0.189	0.0354
all	0.540	0.0999
twmr	0.434	0.0830
ptwas	0.356	0.0650
mrlocus	0.295	0.0530
ecaviar-mrlocus	0.299	0.0569
lda-mr-egger	0.682	0.1230
pmr-sum-egger	2.180	0.3970

Fig J. Accuracy and interval coverage of methods on the simulation of partial mediation with horizontal pleiotropy, including LDA-MR-Egger and PMR-Summary-Egger. As PMR-Summary-Egger does not provide a standard error for the causal effect, interval coverage was only computed for LDA-MR-Egger.

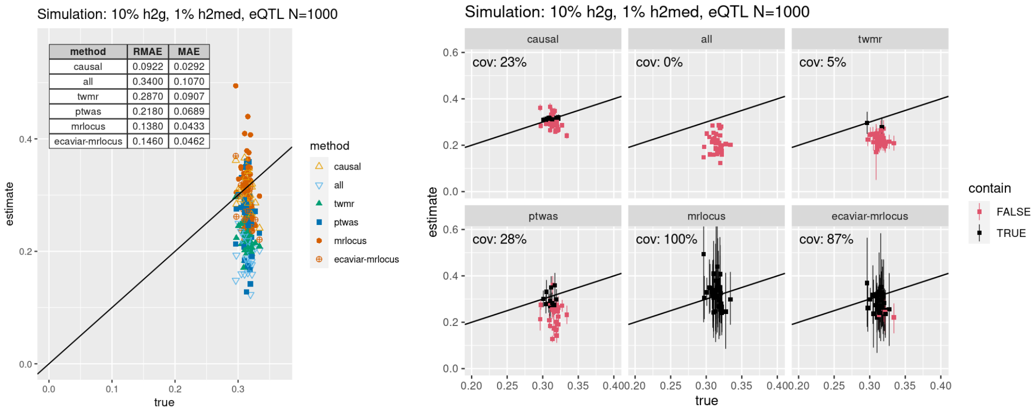

method	RMAE	MAE
causal	0.0922	0.0292
all	0.3400	0.1070
twmr	0.2870	0.0907
ptwas	0.2180	0.0689
mrlocus	0.1380	0.0433
ecaviar-mrlocus	0.1460	0.0462

Fig K. Accuracy and interval coverage of TWMR, PTWAS, MRLocus, and eCAVIAR-MRLocus on the same simulation settings as simulation A but with increased $N_{\text{eQTL}} = 1000$ (instead of the default value $N_{\text{eQTL}} = 500$).

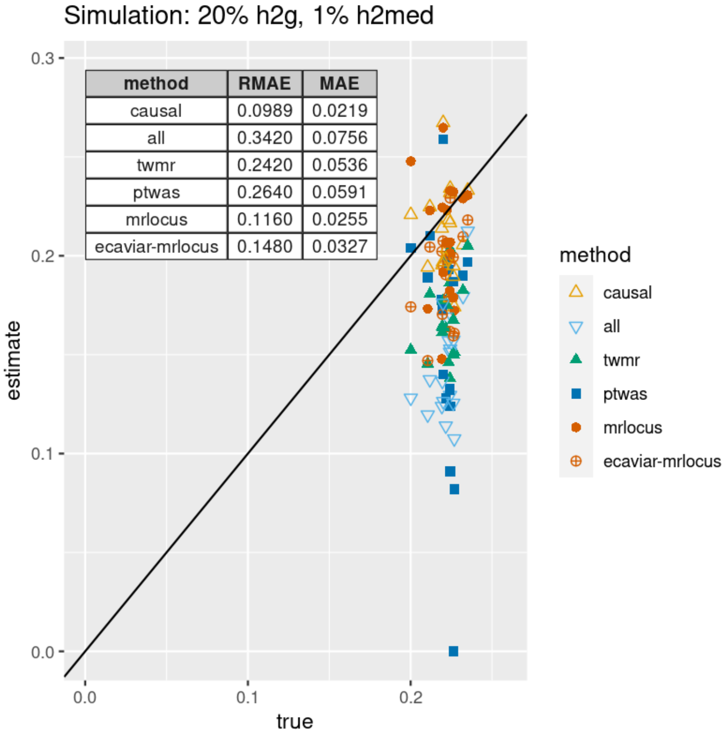

method	RMAE	MAE
causal	0.0989	0.0219
all	0.3420	0.0756
twmr	0.2420	0.0536
ptwas	0.2640	0.0591
mrlocus	0.1160	0.0255
ecaviar-mrlocus	0.1480	0.0327

Fig L. Accuracy of gene-to-trait effect estimation in simulation B.

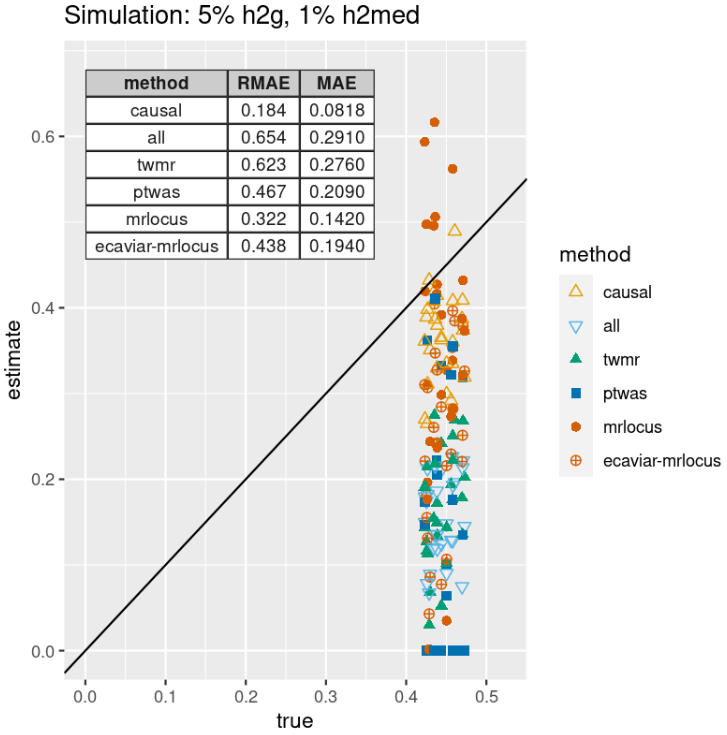

method	RMAE	MAE
causal	0.184	0.0818
all	0.654	0.2910
twmr	0.623	0.2760
ptwas	0.467	0.2090
mrlocus	0.322	0.1420
ecaviar-mrlocus	0.438	0.1940

Fig M. Accuracy of gene-to-trait effect estimation in simulation C.

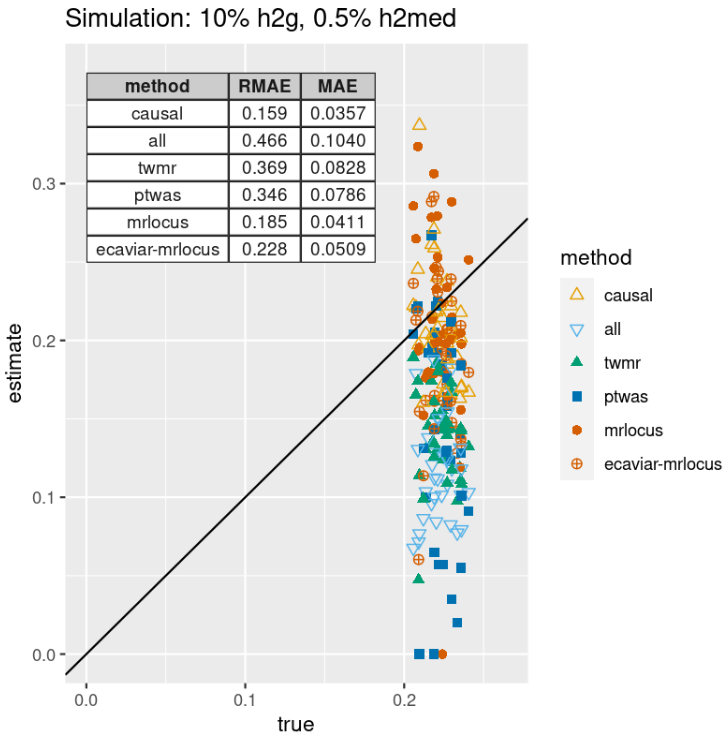

Fig N. Accuracy of gene-to-trait effect estimation in simulation D.

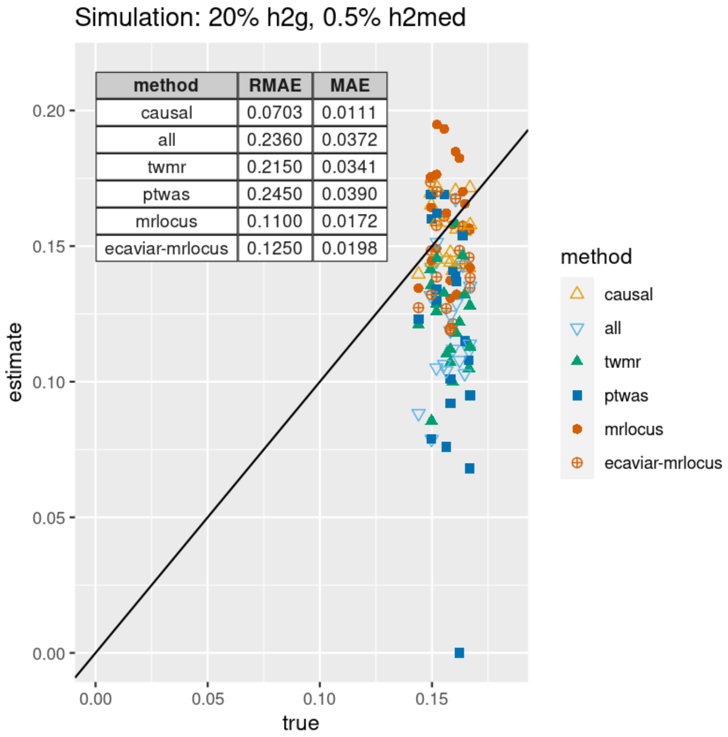

Fig O. Accuracy of gene-to-trait effect estimation in simulation E.

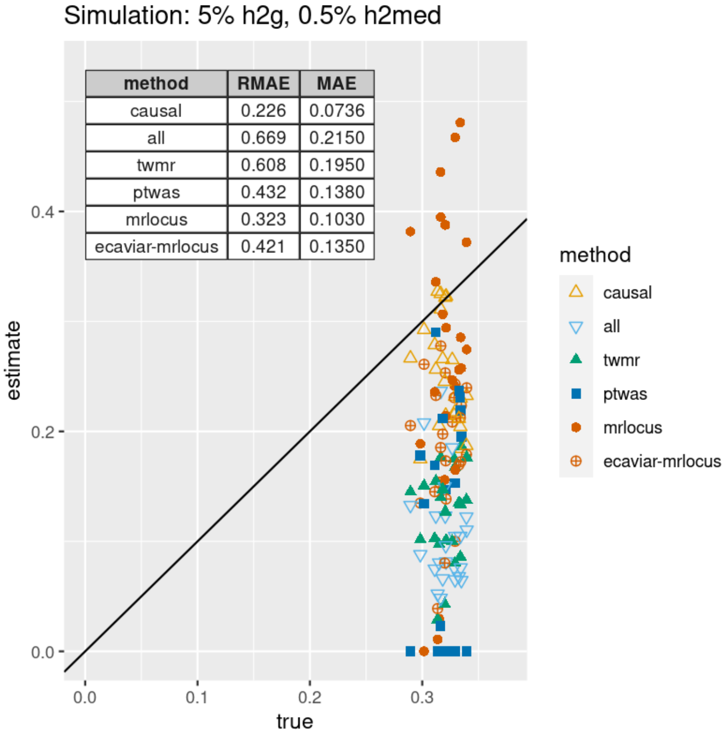

Fig P. Accuracy of gene-to-trait effect estimation in simulation F.

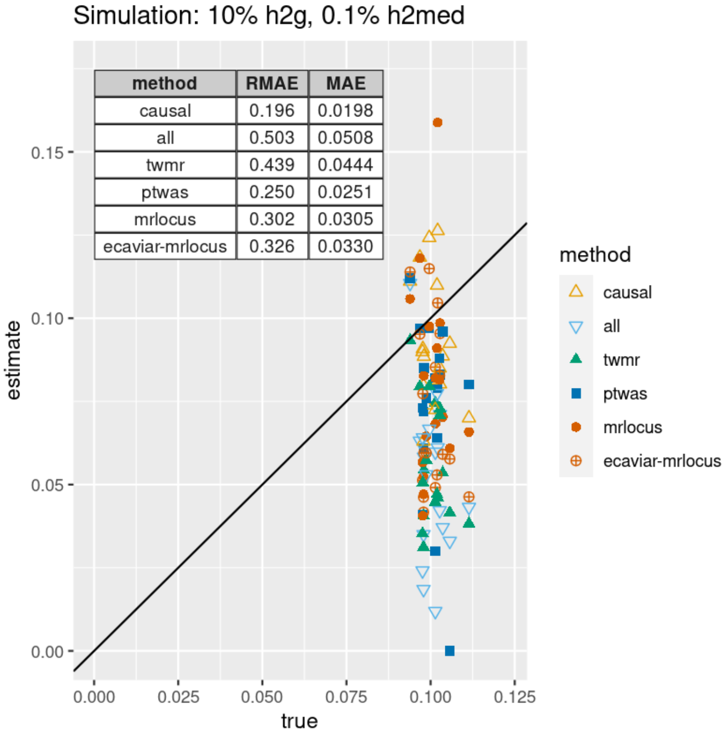

Fig Q. Accuracy of gene-to-trait effect estimation in simulation G.

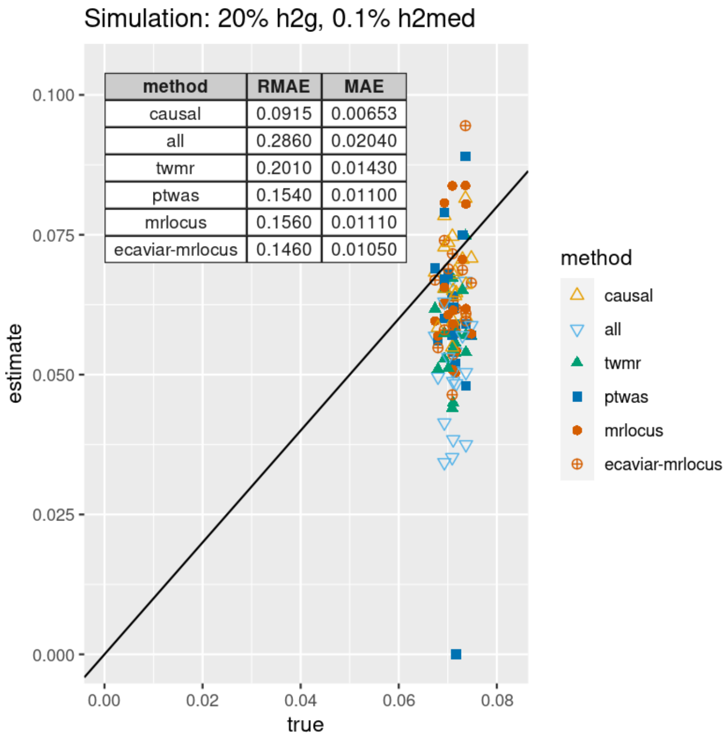

Fig R. Accuracy of gene-to-trait effect estimation in simulation H.

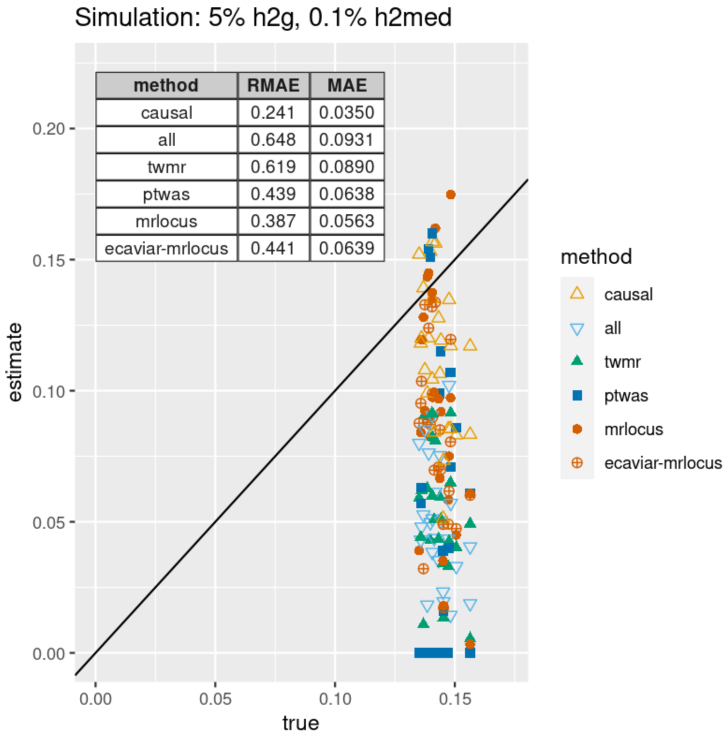

Fig S. Accuracy of gene-to-trait effect estimation in simulation I.

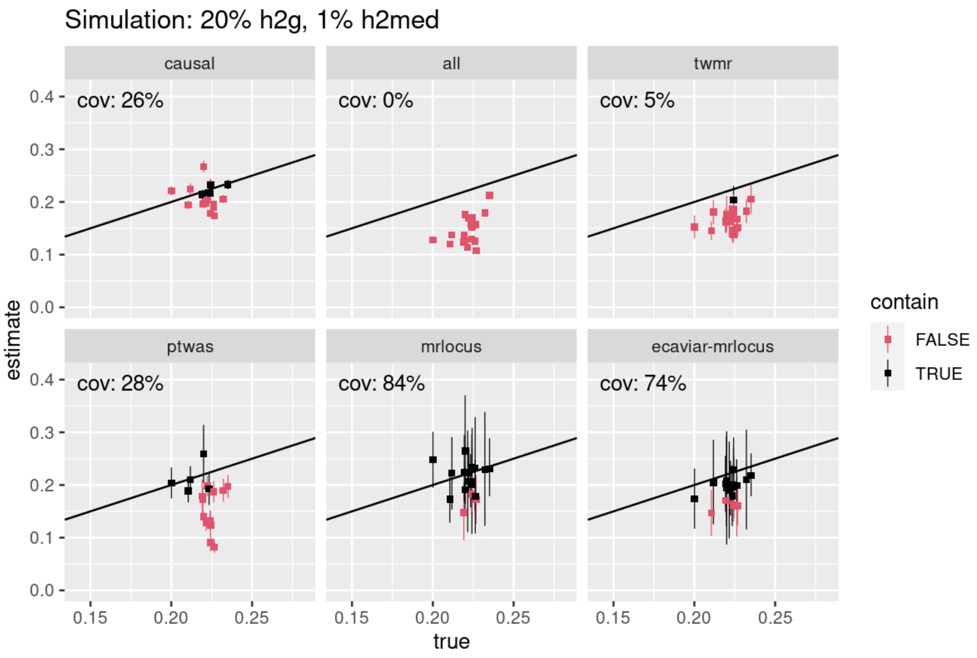

Fig T. Coverage of confidence or credible intervals for the gene-to-trait effect in simulation B.

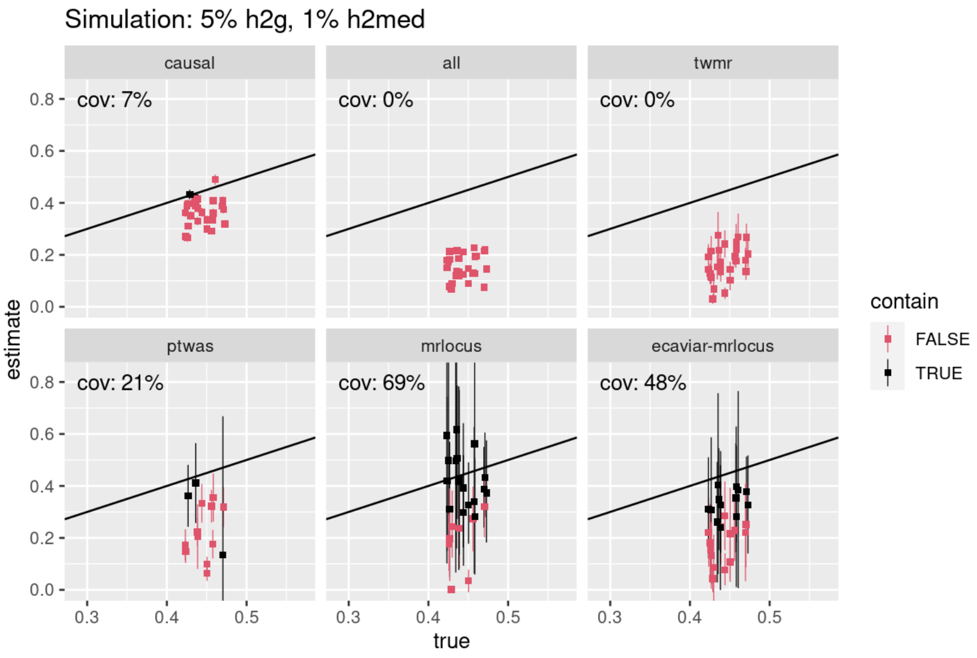

Fig U. Coverage of confidence or credible intervals for the gene-to-trait effect in simulation C.

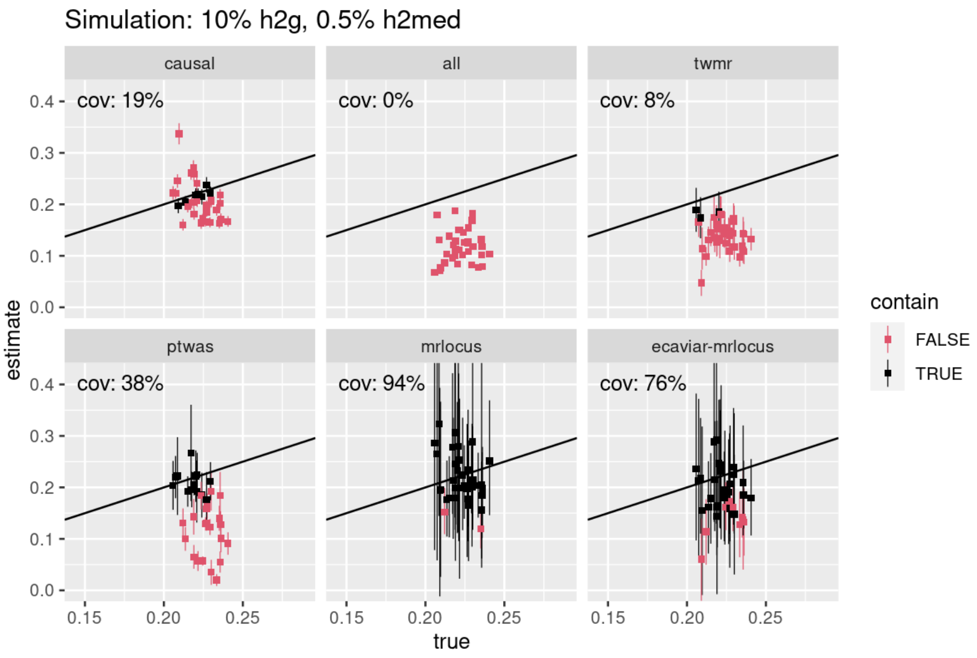

Fig V. Coverage of confidence or credible intervals for the gene-to-trait effect in simulation D.

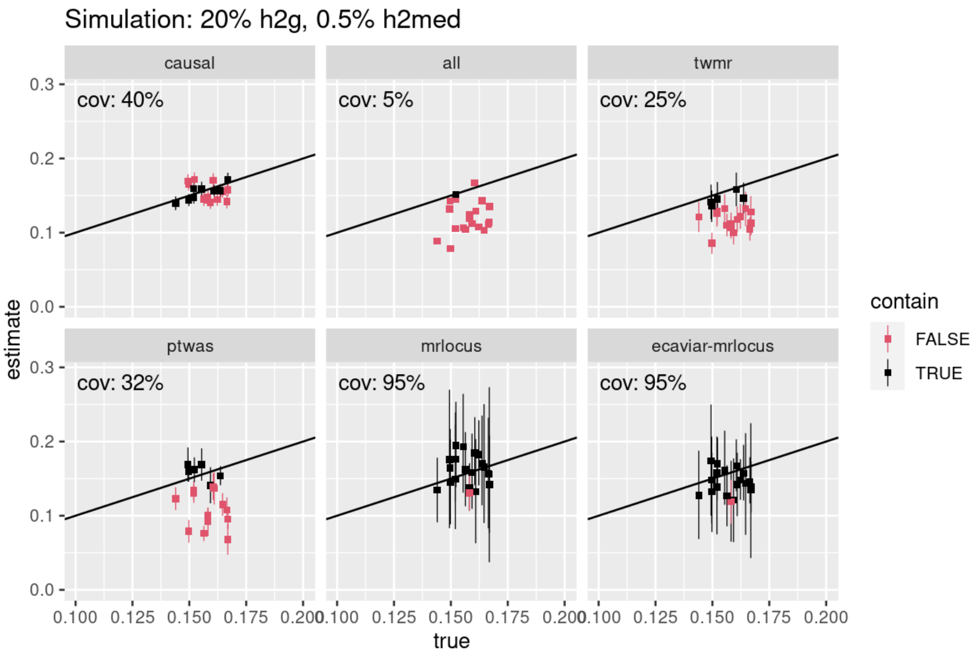

Fig W. Coverage of confidence or credible intervals for the gene-to-trait effect in simulation E.

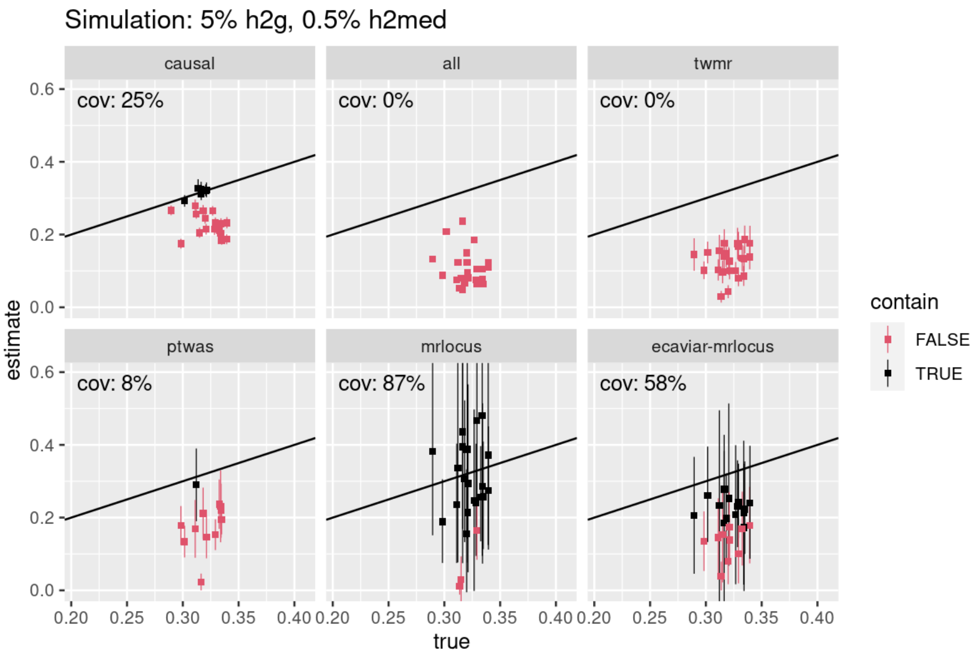

Fig X. Coverage of confidence or credible intervals for the gene-to-trait effect in simulation F.

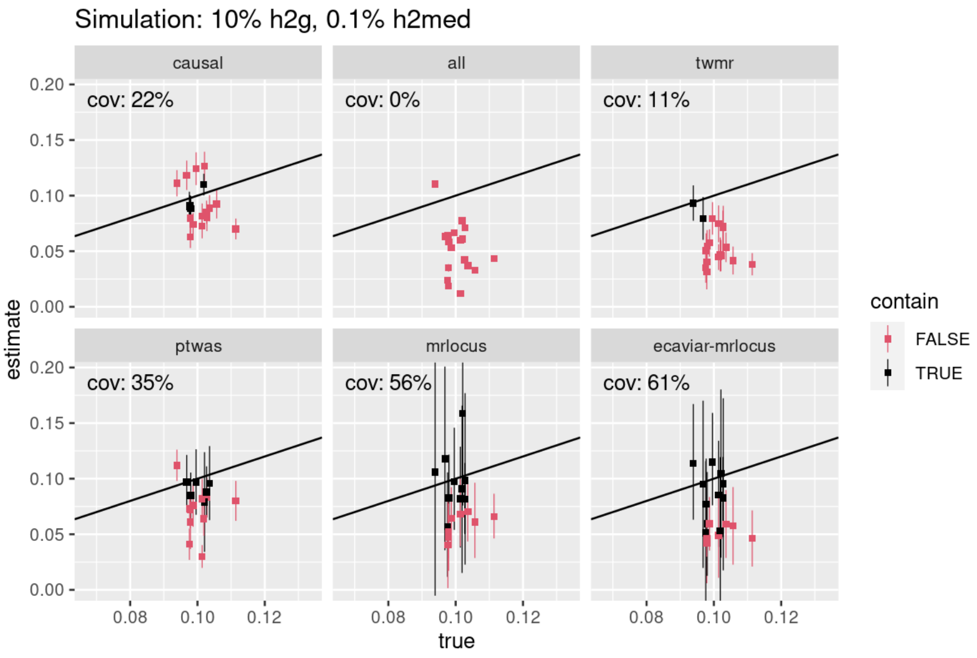

Fig Y. Coverage of confidence or credible intervals for the gene-to-trait effect in simulation G.

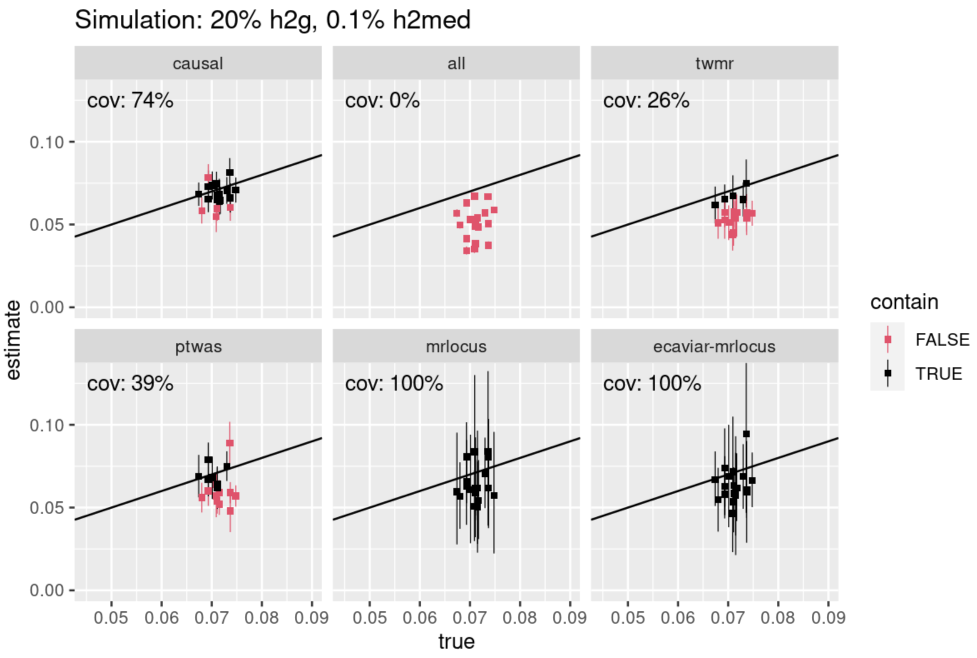

Fig Z. Coverage of confidence or credible intervals for the gene-to-trait effect in simulation H.

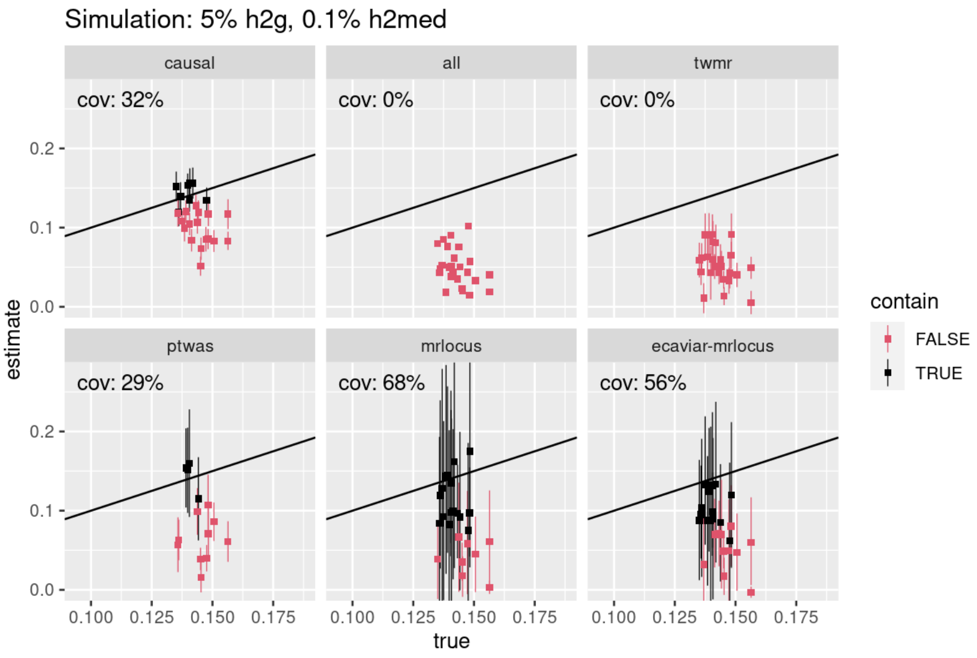

Fig AA. Coverage of confidence or credible intervals for the gene-to-trait effect in simulation I.

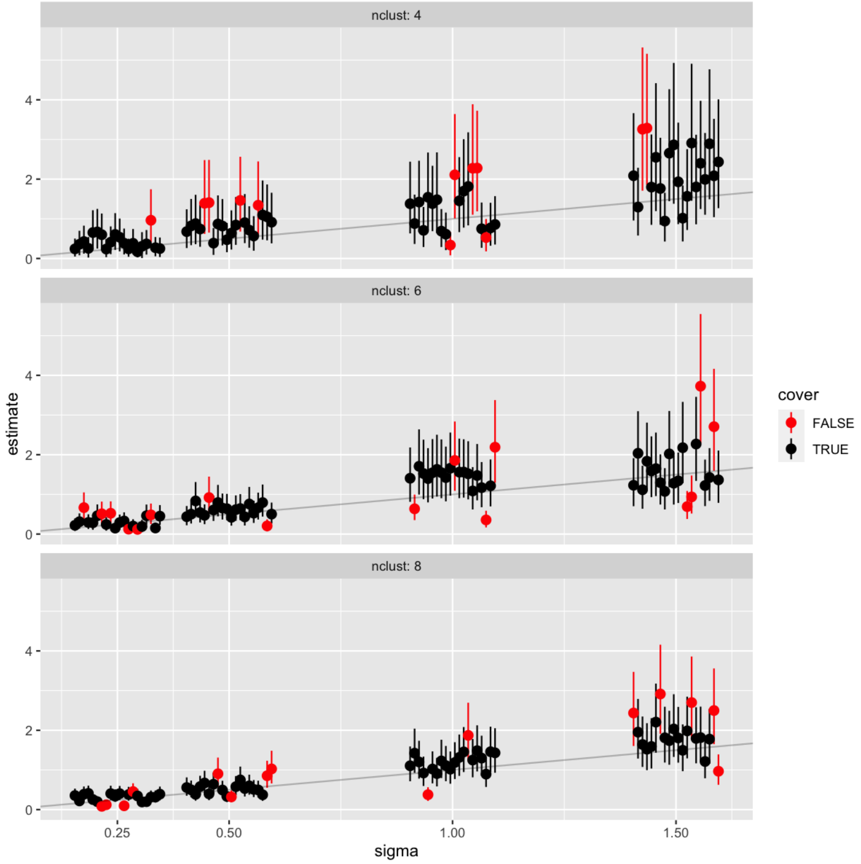

Fig AB. Simulation assessing MRLocus' estimation of the scale of dispersion (σ) across independent signal clusters. The summary statistics for eQTL and GWAS were generated from a multivariate normal distribution as in the eCAVIAR model, using a simulated LD matrix. The true slope (α) was set to 1, the true σ varied among the values $\{0.25, 0.5, 1, 1.5\}$ (x-axis), and the number of LD-independent clusters varied between 4, 6, and 8 (top, middle, bottom panels), with 20 iterations per setting (plotted with horizontal spacing to avoid overplotting). The posterior mean is indicated with a dot, while 80% quantile-based credible intervals and their coverage of the true value are indicated with the line and its color. The simulation script is included within the MRLocus package test directory, as an R script `"test_sigma.R"`.

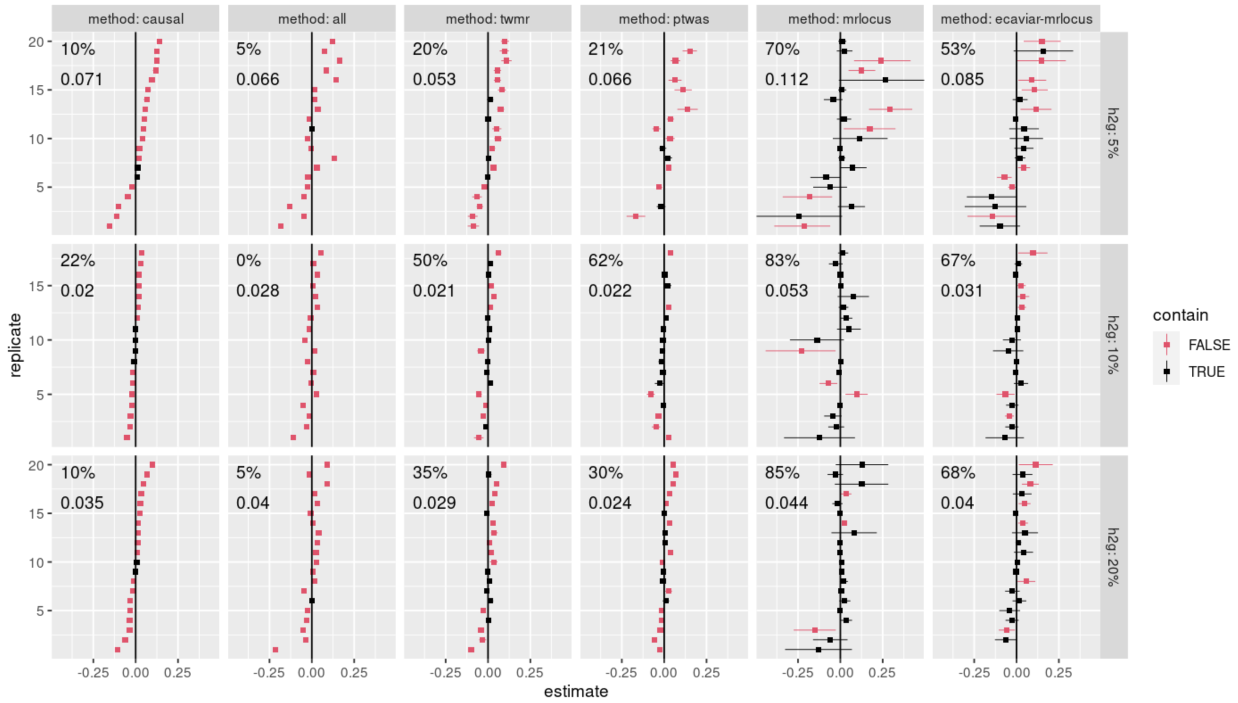

Fig AC. Coverage of confidence or credible intervals for the 3 null simulation settings.

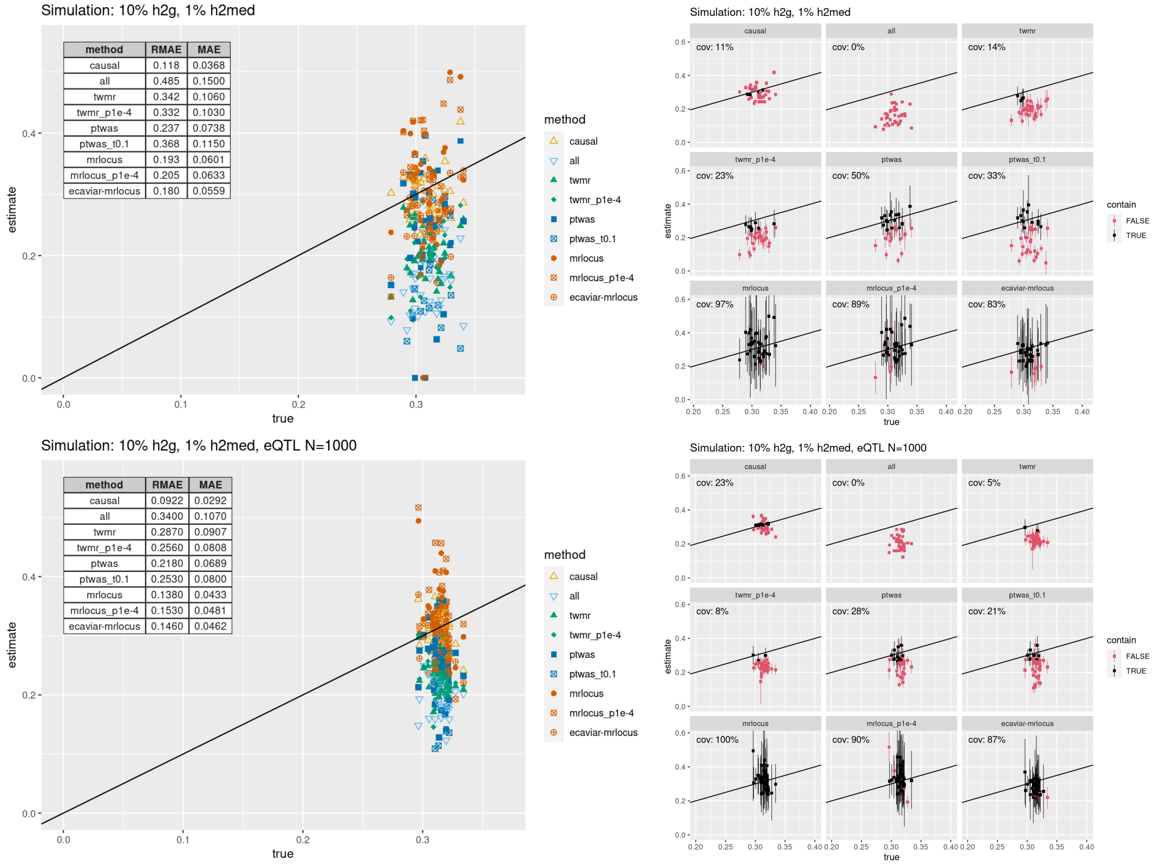

Fig AD. Assessment of methods at eQTL $N = 500$ and $N = 1000$ at alternative thresholds: p-value 0.0001 for PLINK clumping (labeled `_p1e-4`) and using a more lenient PIP threshold of 0.1 (labeled `_t0.1`).

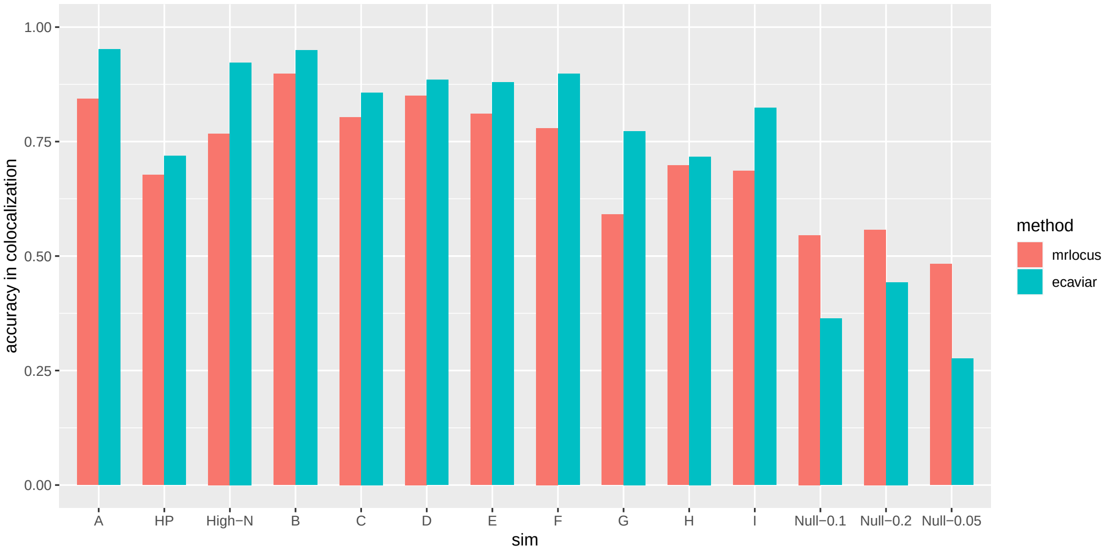

Fig AE. Assessment of colocalization accuracy across simulation settings. Shown is the ratio of correctly identified causal eSNPs for all signal clusters containing a true causal eSNP, aggregating across all iterations. Additionally, if the method identified a SNP with correlation of > 0.95 to the true causal eSNP, it was counted as a correct identification.

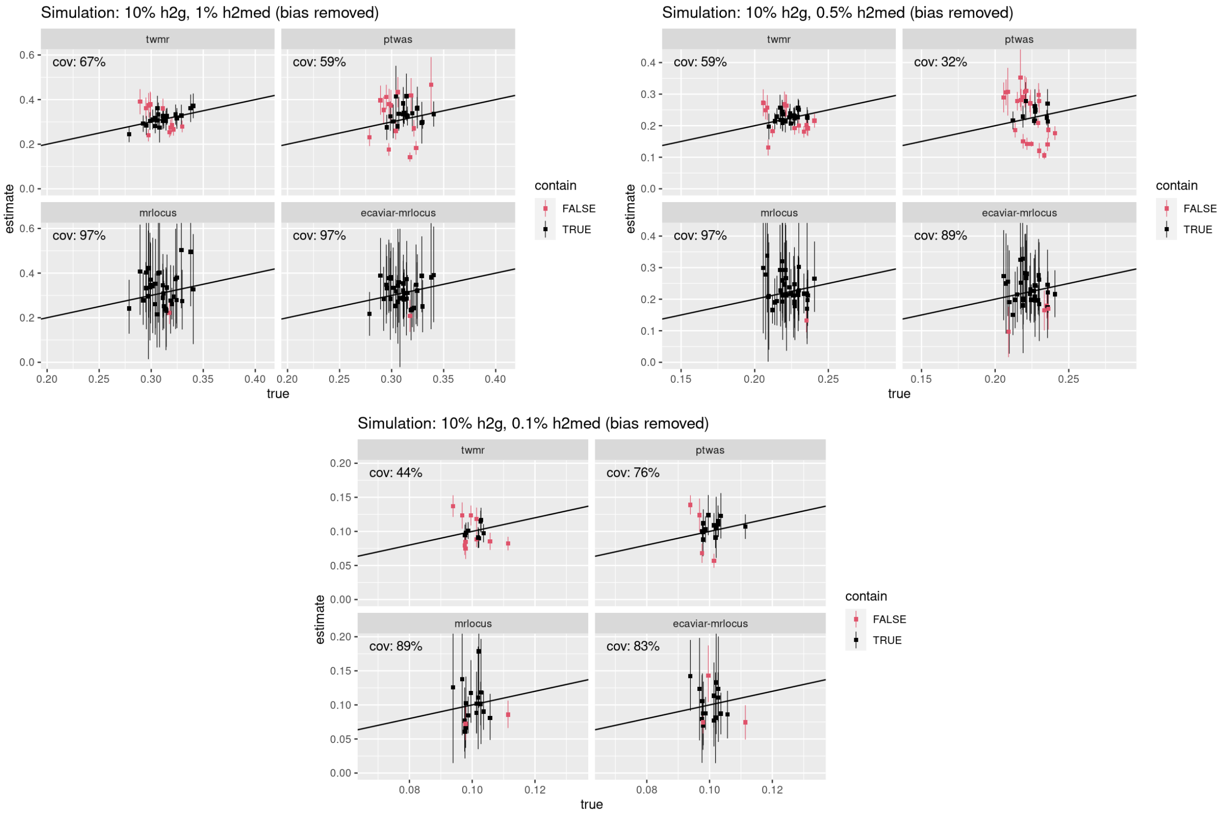

Fig AF. Coverage of confidence and credible intervals after removing estimator bias using the sample average for the estimator and information about the true population-level gene-to-trait effect. Bias was removed and coverage recalculated for simulations with $h^2g = 10\%$: simulations A, D and G. For TWMR, PTWAS, MRLocus, and eCAVIAR-MRLocus, the bias in simulation A was $\{-0.112, -0.079, -0.004, -0.052\}$ respectively. For simulation D the bias was $\{-0.083, -0.086, -0.014, -0.037\}$, and for simulation G the bias was $\{-0.044, -0.027, -0.020, -0.028\}$.

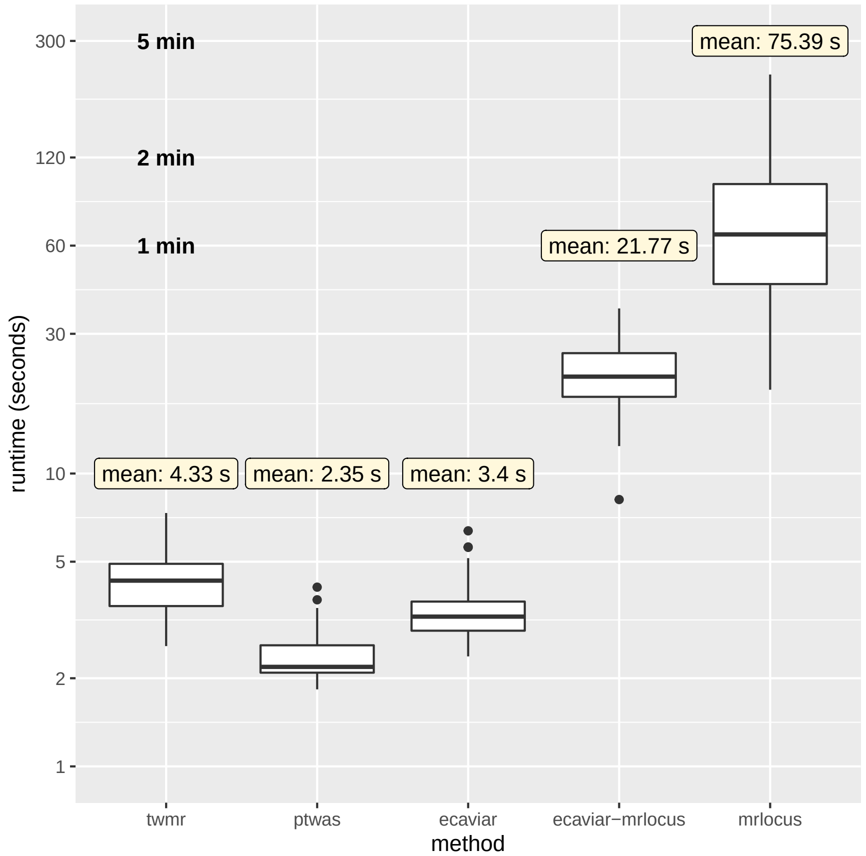

Fig AG. Runtime for TWMR, PTWAS, and MRLocus on the 80 iterations from simulation A and eQTL $N = 1000$ simulation. The runtime for a single locus is shown on the y-axis (log scale), with MRLocus colocalization using 4 cores. "eCAVIAR-MRLocus" indicates the time for MRLocus slope fitting following eCAVIAR colocalization, while "MRLocus" indicates the time for both colocalization and slope fitting.

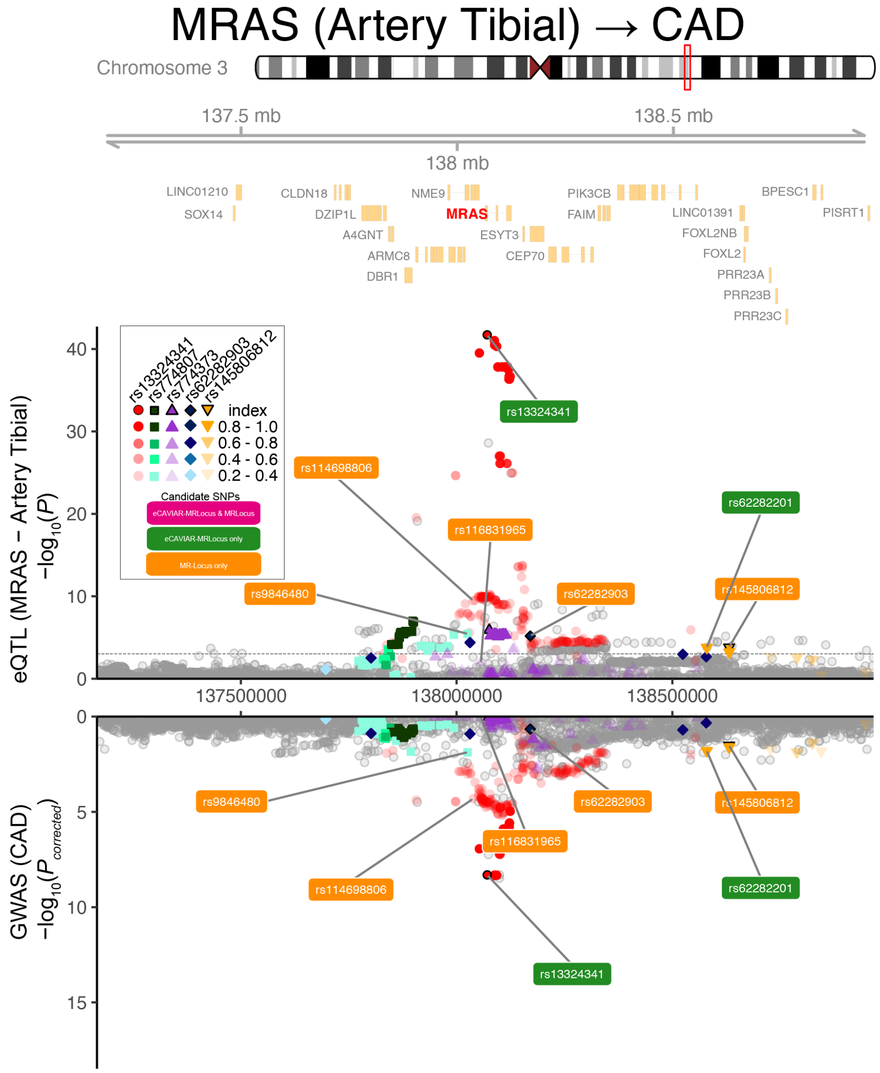

Fig AH. Colocalized signals in the *MRAS* region. From top panel to bottom, gene model (NCBI Refseq), eQTL for MRAS in artery tibial (GTEx; N = 663) and CAD association within CARDIoGRAMplusC4D ($N_{\text{case}} = 60{,}801$ and $N_{\text{control}} = 123{,}504$) (M. Nikpay et al., 2015). LD was calculated to nearly-LD-independent index SNPs within 1KG EUR and colored accordingly. Dashed line indicates a significance threshold at p = 0.001 or p = $5 \text{x} 10^{-8}$ for eQTL and GWAS respectively. Colored labels indicate eSNPs used for slope fitting with both methods, eCAVIAR-MRLocus, or MRLocus.

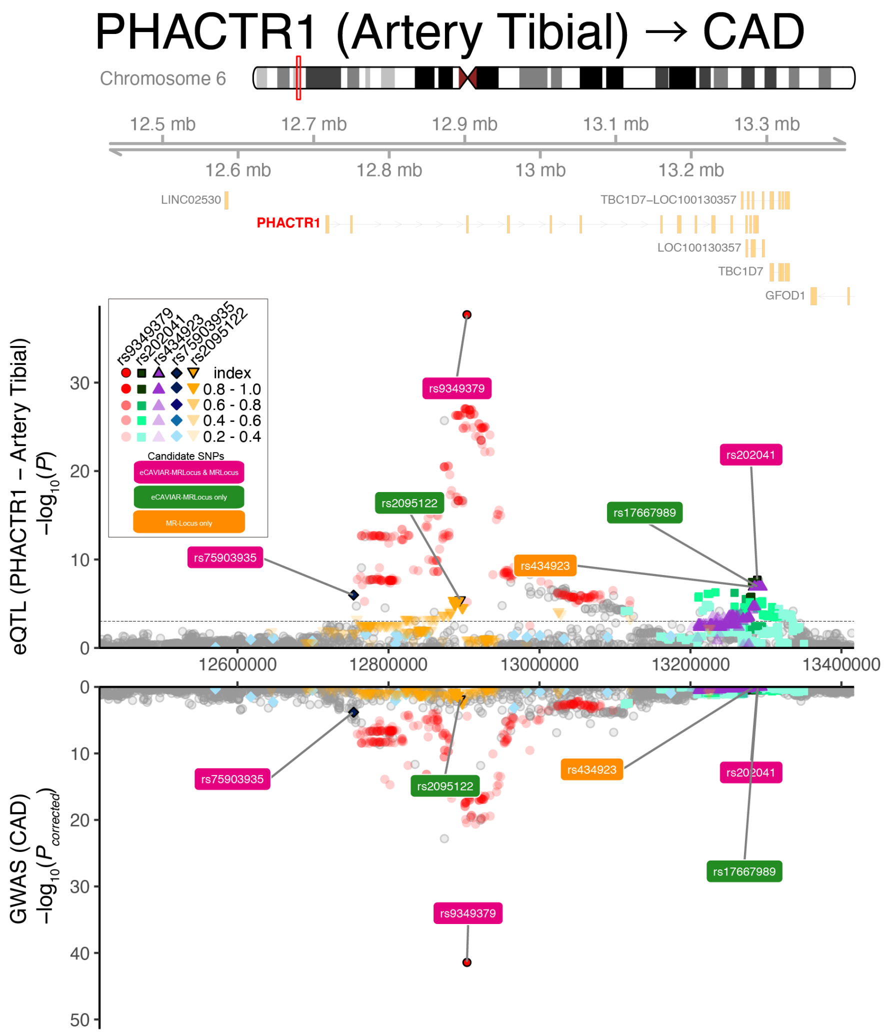

Fig AI. Colocalized signals in the *PHACTR1* region. From top panel to bottom, gene model (NCBI Refseq), eQTL for PHACTR1 in artery tibial (GTEx; N = 663) and CAD association within CARDIoGRAMplusC4D ($N_{\text{case}} = 60{,}801$ and $N_{\text{control}} = 123{,}504$) (M. Nikpay et al., 2015). LD was calculated to nearly-LD-independent index SNPs within 1KG EUR and colored accordingly. Dashed line indicates a significance threshold at p = 0.001 or p = $5\text{x}10^{-8}$ for eQTL and GWAS respectively. Colored labels indicate eSNPs used for slope fitting with both methods, eCAVIAR-MRLocus, or MRLocus.

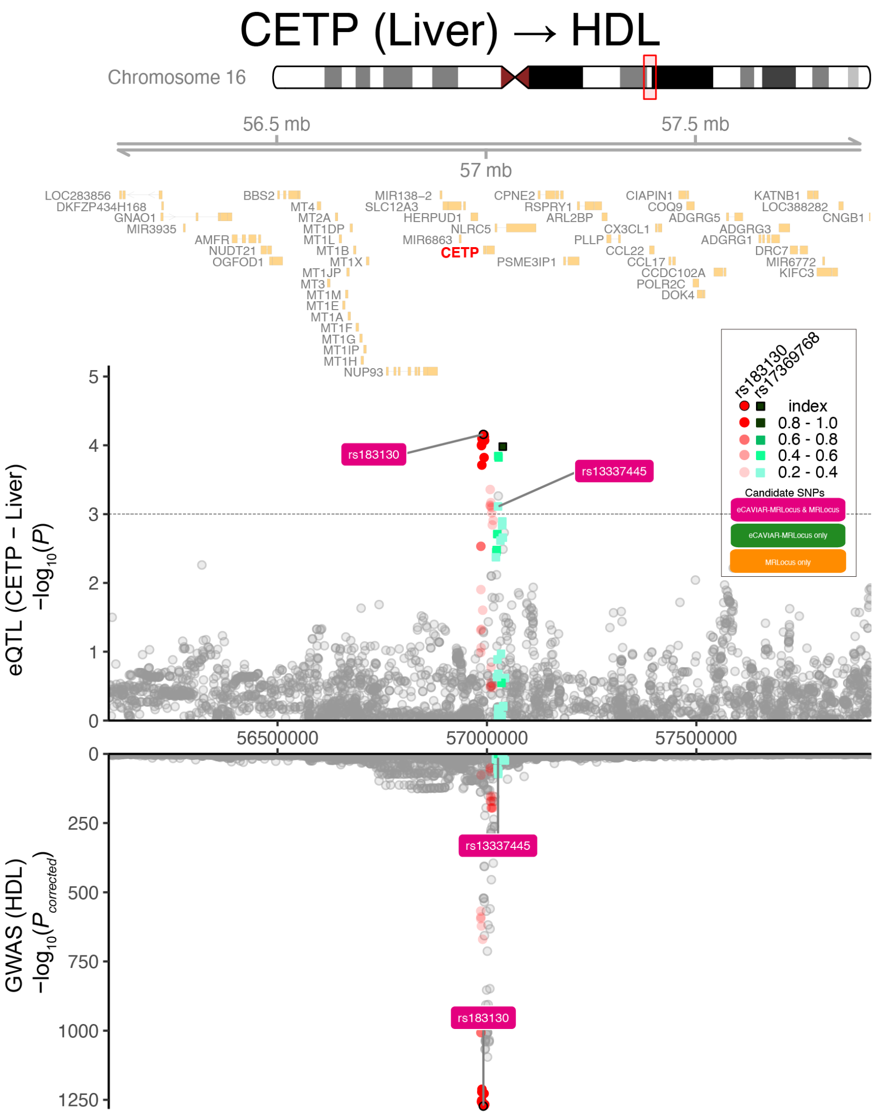

Fig AJ. Colocalized signals in the *CETP* region. From top panel to bottom, gene model (NCBI Refseq), eQTL for CETP in liver (N = 588) (Strunz et al., 2018) and HDL association within UKBB (N = 315,133). LD was calculated to nearly-LD-independent index SNPs within 1KG EUR and colored accordingly. Dashed line indicates a significance threshold at p = 0.001 or p = $5x10^{-8}$ for eQTL and GWAS respectively. Colored labels indicate eSNPs used for slope fitting with both methods, eCAVIAR-MRLocus, or MRLocus.

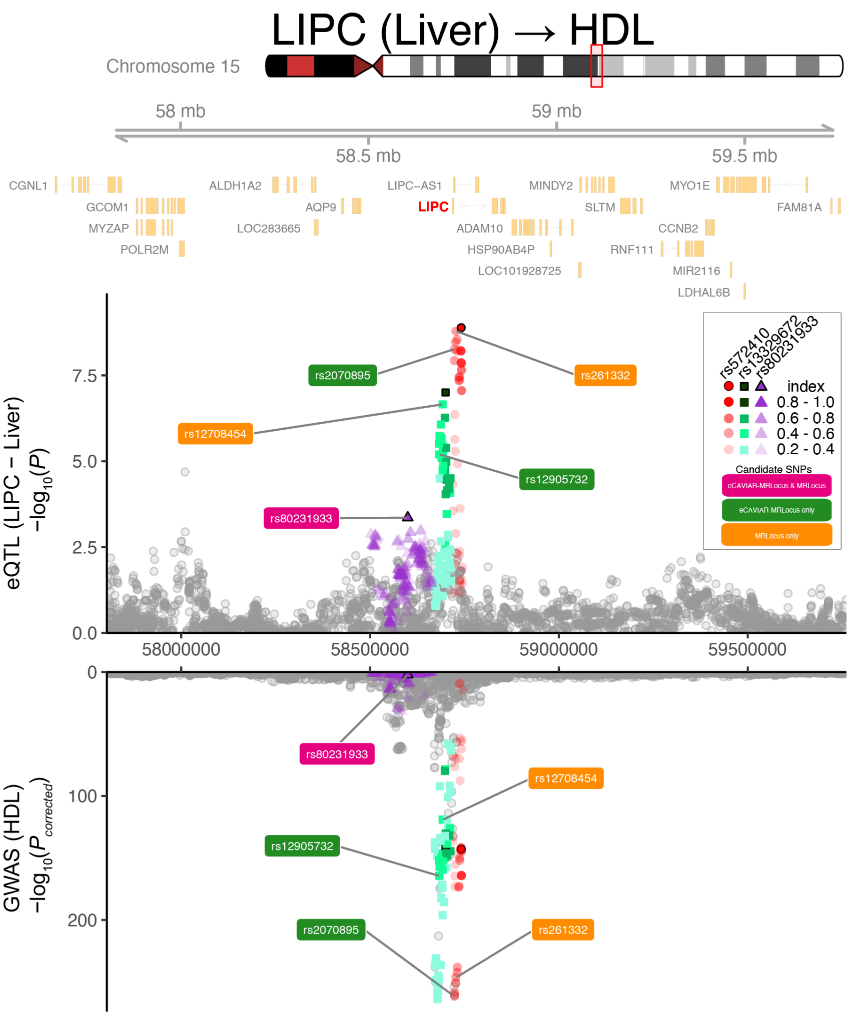

Fig AK. Colocalized signals in the *LIPC* region for liver eQTL. From top panel to bottom, gene model (NCBI Refseq), eQTL for LIPC in liver (N = 588) (Strunz et al., 2018) and HDL association within UKBB (N = 315,133). LD was calculated to nearly-LD-independent index SNPs within 1KG EUR and colored accordingly. Dashed line indicates a significance threshold at p = 0.001 or p = $5x10^{-8}$ for eQTL and GWAS respectively. Colored labels indicate eSNPs used for slope fitting with both methods, eCAVIAR-MRLocus, or MRLocus.

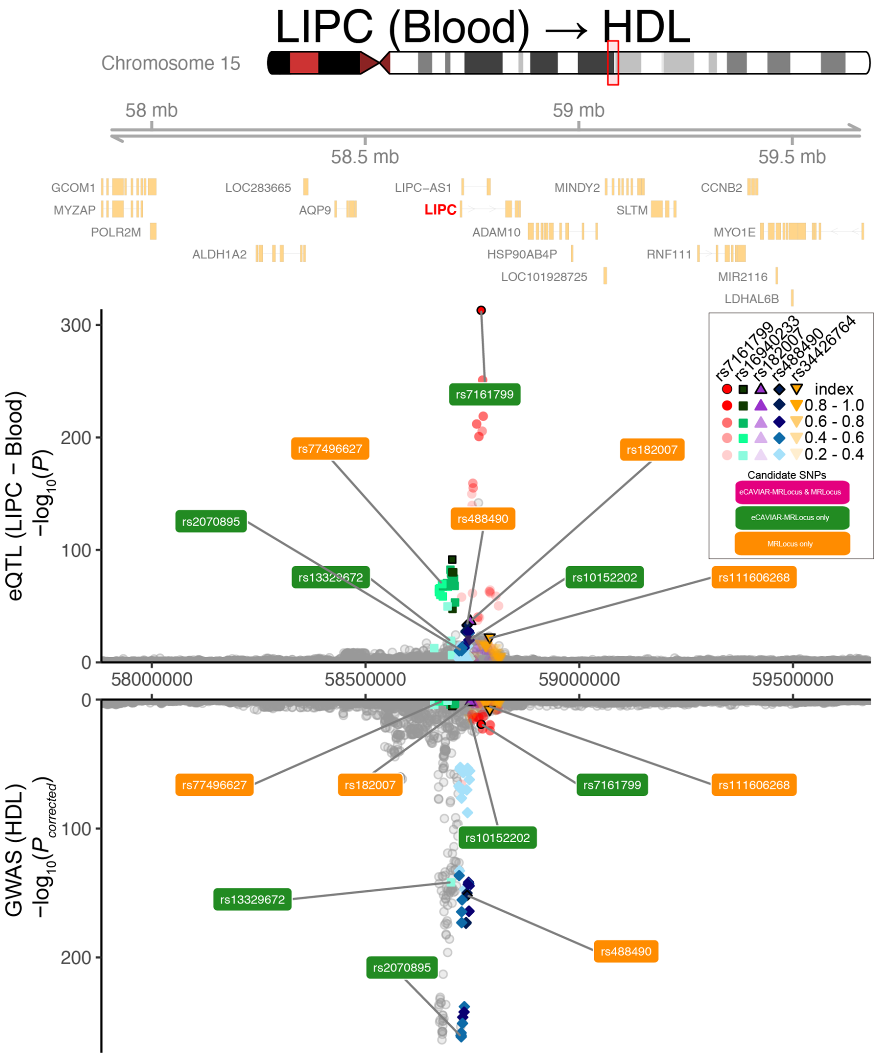

Fig AL. Colocalized signals in the *LIPC* region for blood eQTL. From top panel to bottom, gene model (NCBI Refseq), eQTL for LIPC in blood (N = 31,684) (Vosa et al. 2018) and HDL association within UKBB (N = 315,133). LD was calculated to nearly-LD-independent index SNPs within 1KG EUR and colored accordingly. Dashed line indicates a significance threshold at p = 0.001 or p = 5×10^{-8} for eQTL and GWAS respectively. Colored labels indicate eSNPs used for slope fitting with both methods, eCAVIAR-MRLocus, or MRLocus.

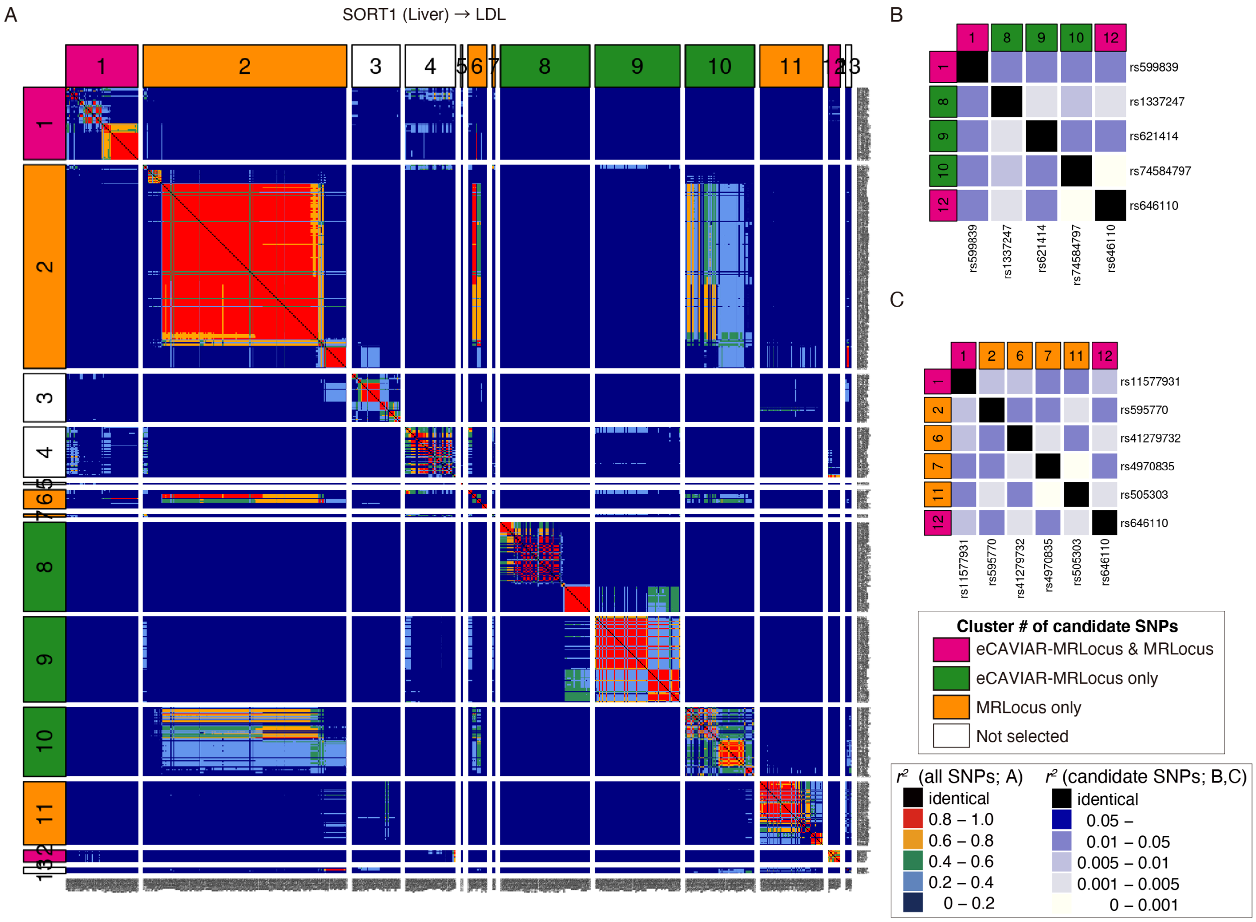

Fig AM. LD pattern across nearly-LD-independent signal clusters from *SORT1* eQTL (liver). (A) r^2 between all SNPs in signal clusters. Color bars at the top and left represent whether the cluster was used for estimation of gene-to-trait effect with both methods, only eCAVIAR-MRLocus, only MRLocus, or neither (trimmed due to pairwise r^2 with other signal clusters). (B) Pairwise r^2 for instruments selected for slope fitting for eCAVIAR-MRLocus. (C) Pairwise r^2 for instruments selected for slope fitting for MRLocus.

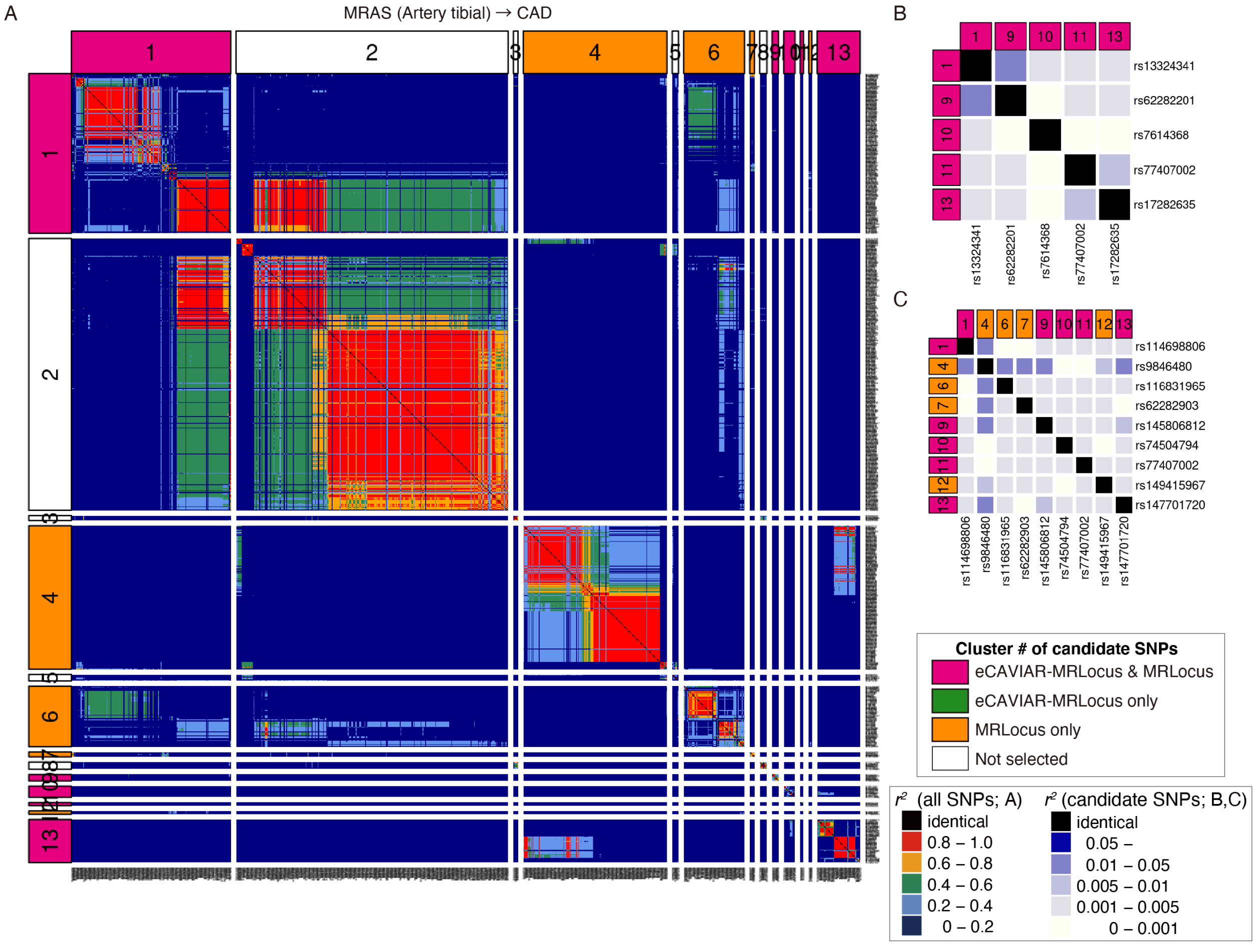

Fig AN. LD pattern across nearly-LD-independent signal clusters from *MRAS* eQTL (artery). (A) r^2 between all SNPs in signal clusters. Color bars at the top and left represent whether the cluster was used for estimation of gene-to-trait effect with both methods, only eCAVIAR-MRLocus, only MRLocus, or neither (trimmed due to pairwise r^2 with other signal clusters). (B) Pairwise r^2 for instruments selected for slope fitting for eCAVIAR-MRLocus. (C) Pairwise r^2 for instruments selected for slope fitting for MRLocus.

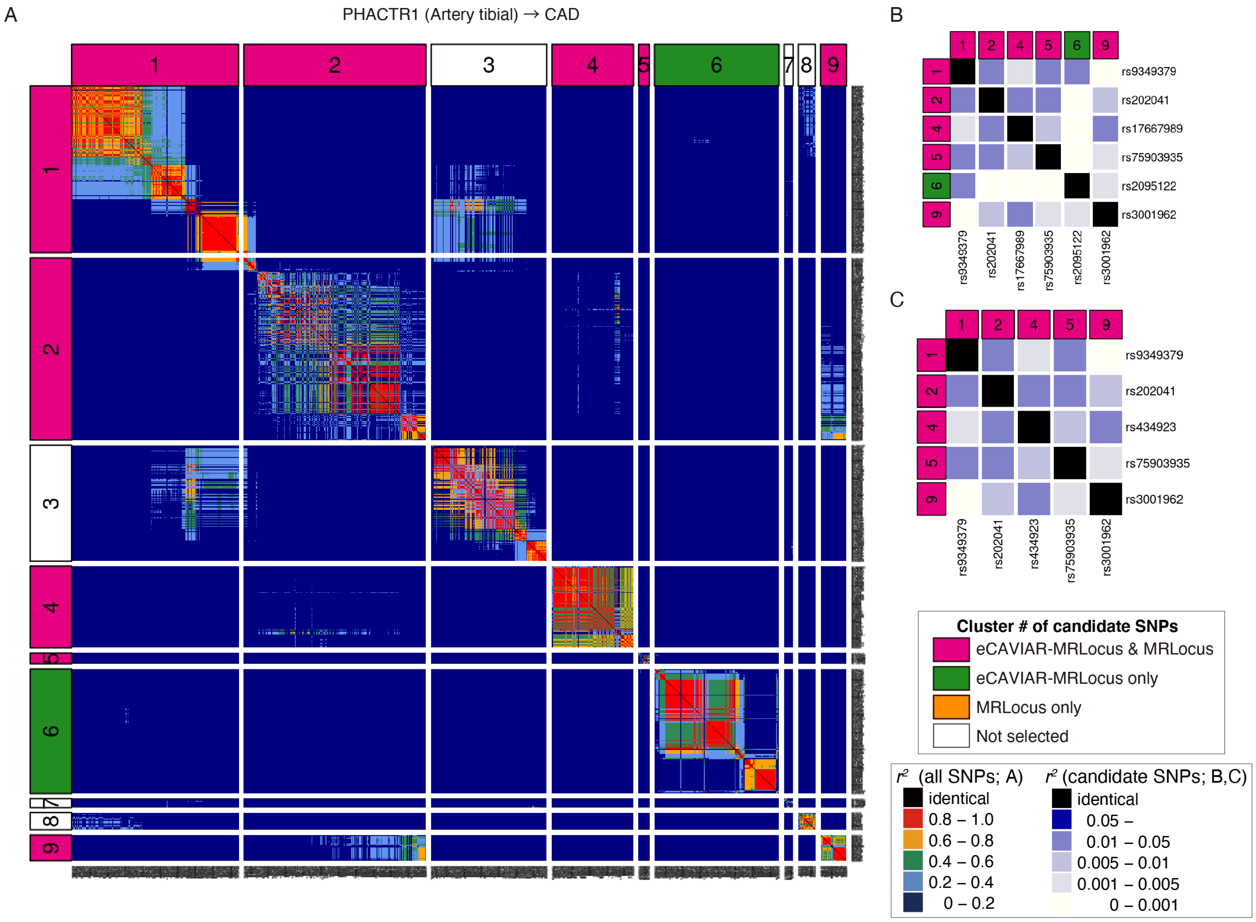

Fig AO. LD pattern across nearly-LD-independent signal clusters from *PHACTR1* eQTL (artery). (A) r^2 between all SNPs in signal clusters. Color bars at the top and left represent whether the cluster was used for estimation of gene-to-trait effect with both methods, only eCAVIAR-MRLocus, only MRLocus, or neither (trimmed due to pairwise r^2 with other signal clusters). (B) Pairwise r^2 for instruments selected for slope fitting for eCAVIAR-MRLocus. (C) Pairwise r^2 for instruments selected for slope fitting for MRLocus.

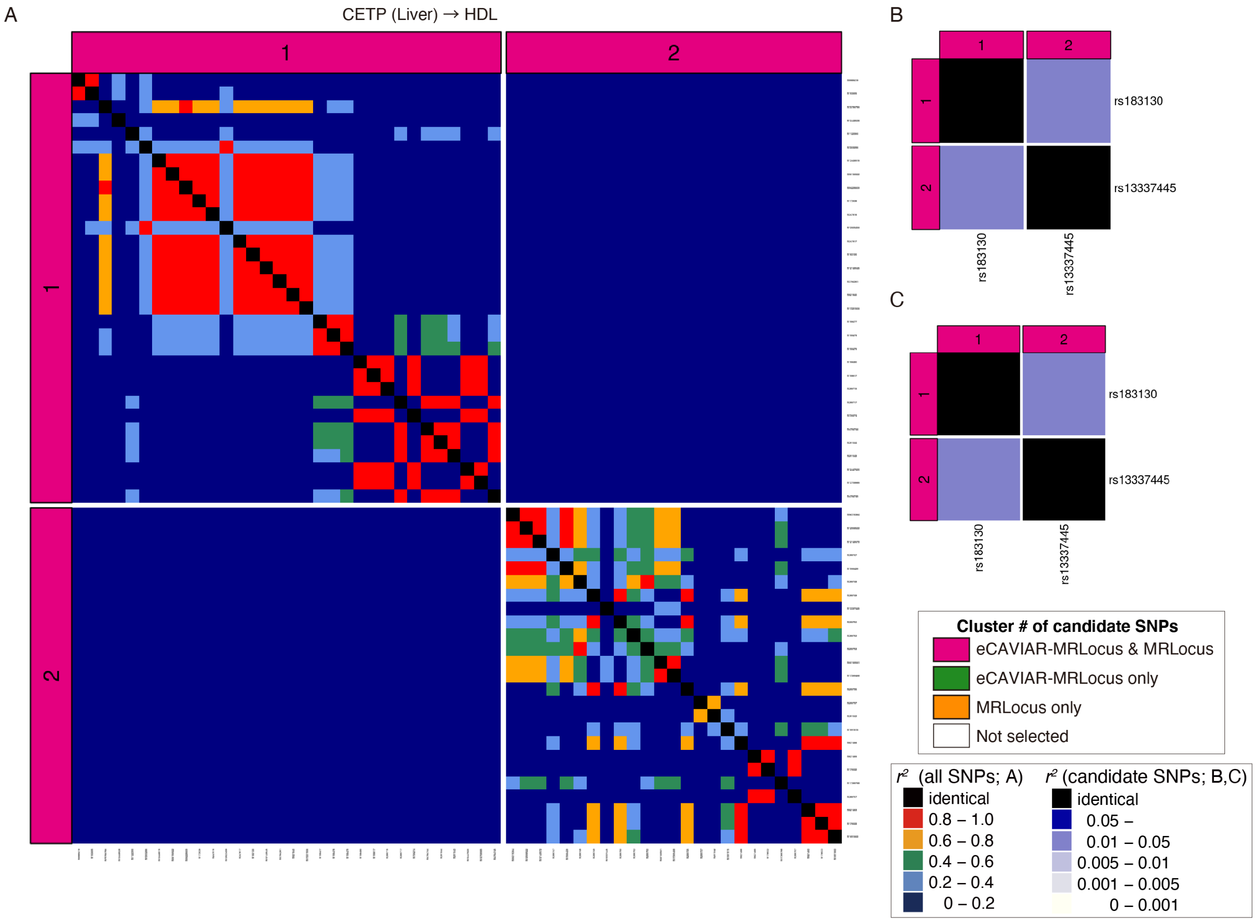

Fig AP. LD pattern across nearly-LD-independent signal clusters from *CETP* eQTL (liver). (A) r^2 between all SNPs in signal clusters. Color bars at the top and left represent whether the cluster was used for estimation of gene-to-trait effect with both methods, only eCAVIAR-MRLocus, only MRLocus, or neither (trimmed due to pairwise r^2 with other signal clusters). (B) Pairwise r^2 for instruments selected for slope fitting for eCAVIAR-MRLocus. (C) Pairwise r^2 for instruments selected for slope fitting for MRLocus.

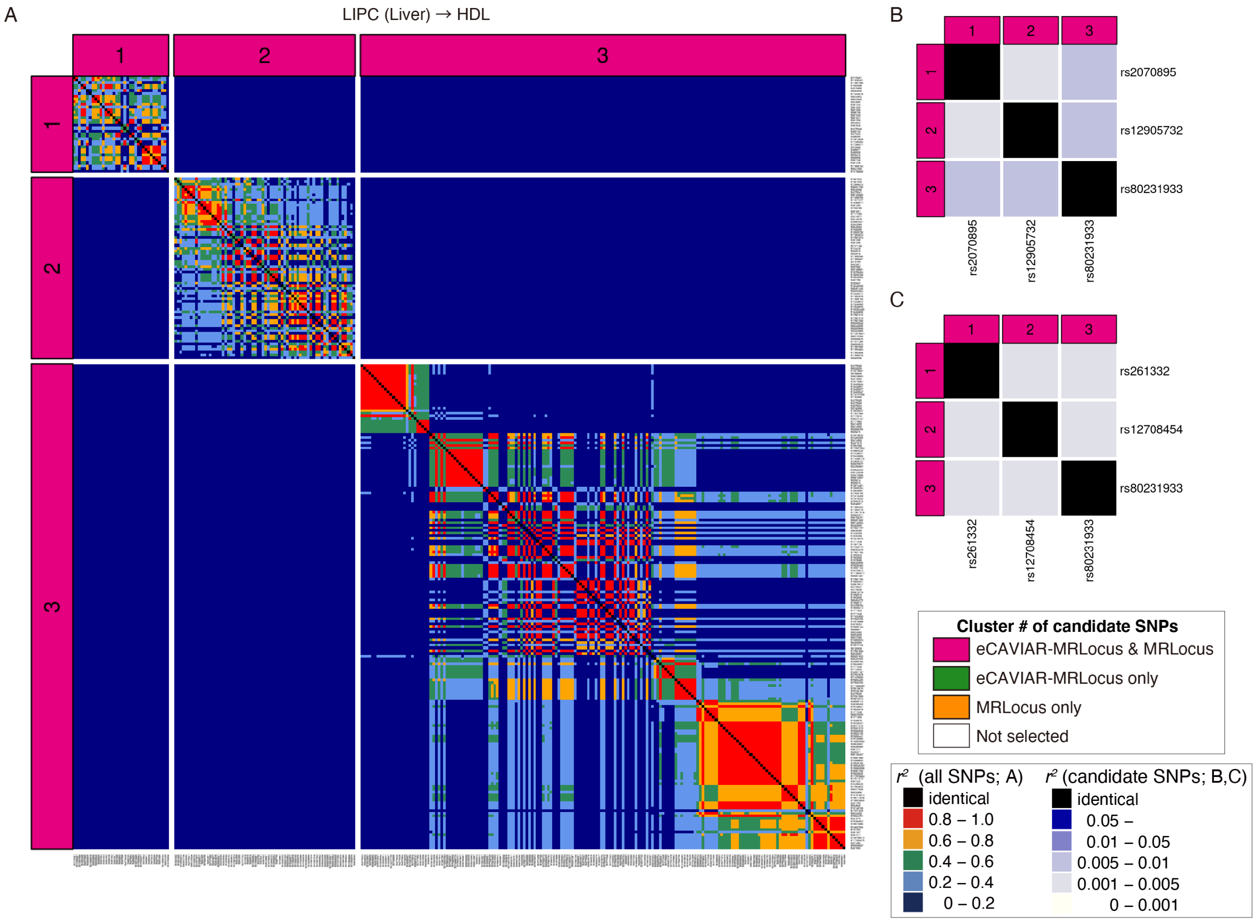

Fig AQ. LD pattern across nearly-LD-independent signal clusters from *LIPC* eQTL (liver). (A) r^2 between all SNPs in signal clusters. Color bars at the top and left represent whether the cluster was used for estimation of gene-to-trait effect with both methods, only eCAVIAR-MRLocus, only MRLocus, or neither (trimmed due to pairwise r^2 with other signal clusters). (B) Pairwise r^2 for instruments selected for slope fitting for eCAVIAR-MRLocus. (C) Pairwise r^2 for instruments selected for slope fitting for MRLocus.

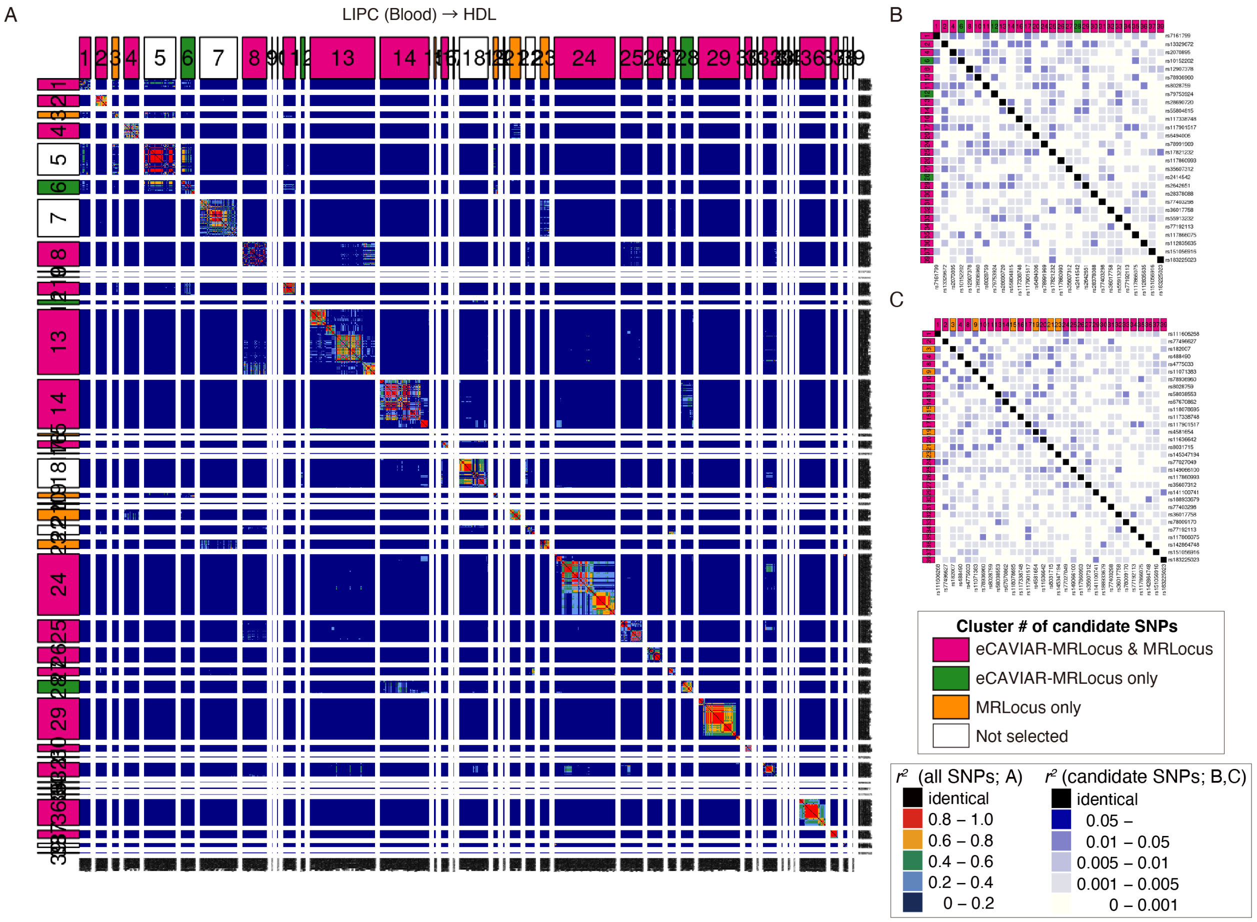

Fig AR. LD pattern across nearly-LD-independent signal clusters from *LIPC* eQTL (blood). (A) r^2 between all SNPs in signal clusters. Color bars at the top and left represent whether the cluster was used for estimation of gene-to-trait effect with both methods, only eCAVIAR-MRLocus, only MRLocus, or neither (trimmed due to pairwise r^2 with other signal clusters). (B) Pairwise r^2 for instruments selected for slope fitting for eCAVIAR-MRLocus. (C) Pairwise r^2 for instruments selected for slope fitting for MRLocus.

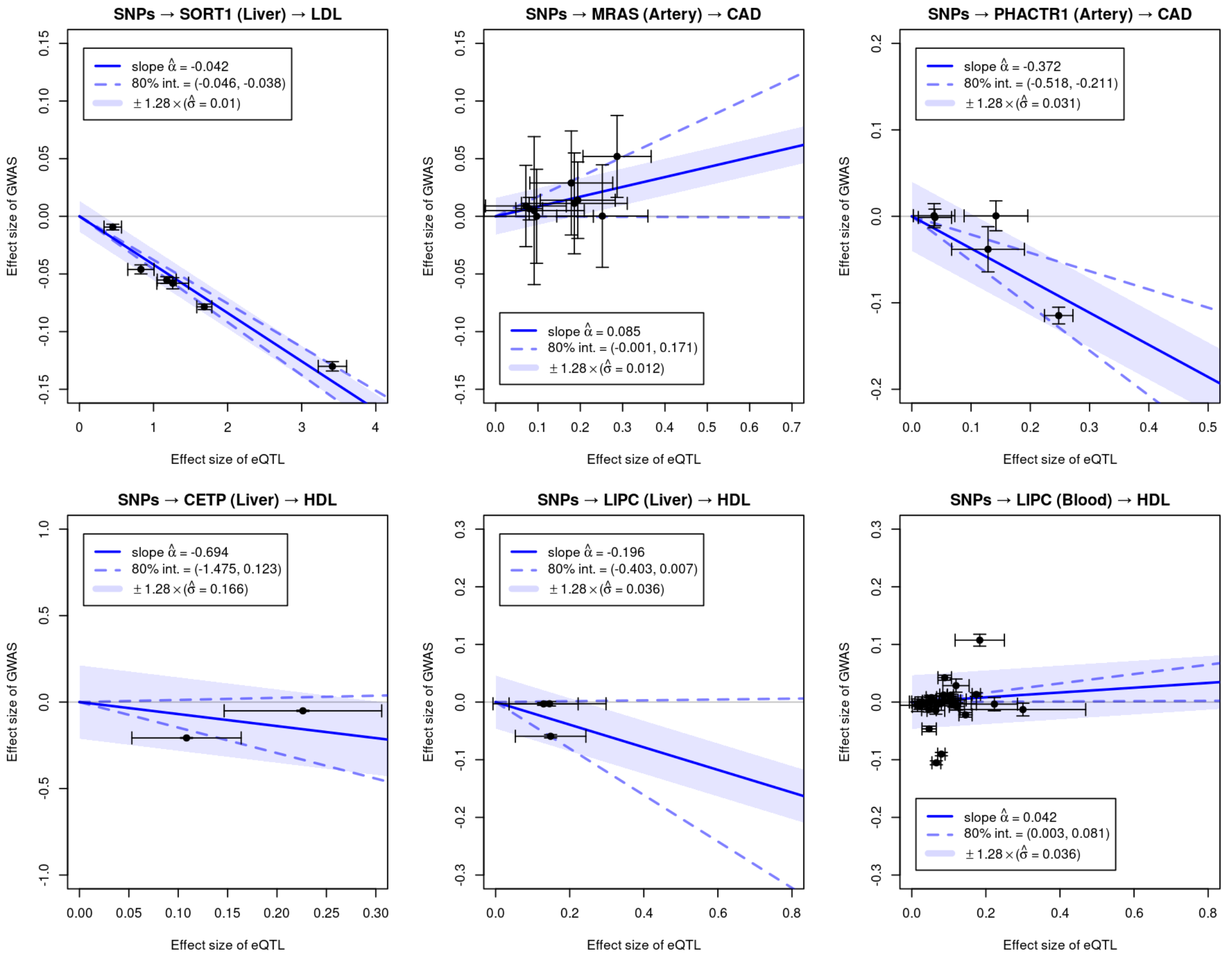

Fig AS. MRLocus plots for six eQTL-GWAS dataset pairs, using MRLocus for colocalization.

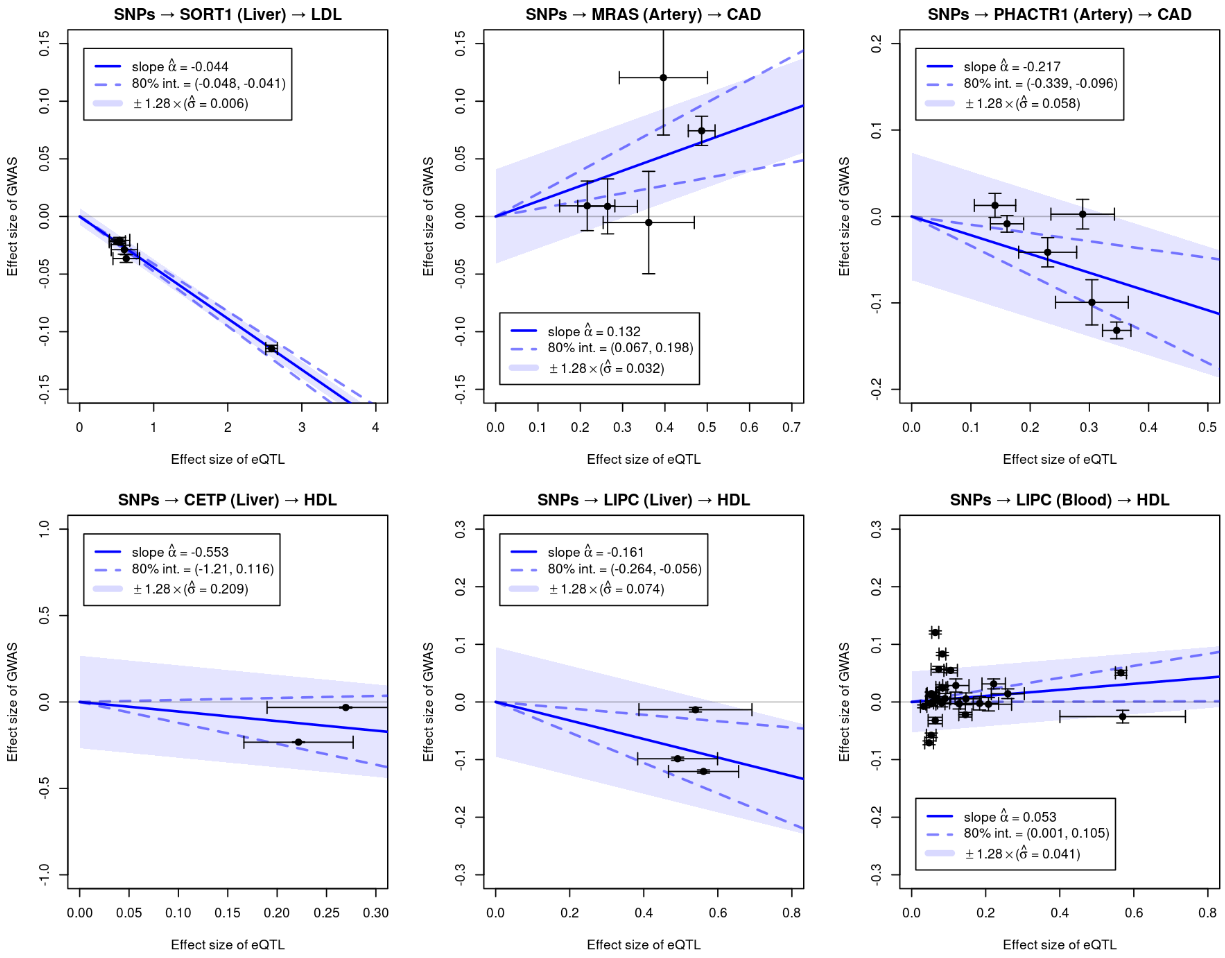

Fig AT. MRLocus plots for six eQTL-GWAS dataset pairs, using eCAVIAR for colocalization.
