# Supplementary material for: MRLocus: Identifying causal genes mediating a trait through Bayesian estimation of allelic heterogeneity
Source: PLoS Genet. 2021 Apr 19;17(4):e1009455. doi: 10.1371/journal.pgen.1009455 (PMC8084342; doi:10.1371/journal.pgen.1009455)
Supplement: Attachment — Submitted filename: Response for MRLocus.pdf [file pgen.1009455.s003.pdf]

January 25, 2021

Response to reviewers for manuscript:

"MRLocus: identifying causal genes mediating a trait through Bayesian estimation of allelic heterogeneity"

We were glad to see that the reviewers agree that, "the MRLocus method devised by these authors is one of the better and innovative approaches in this space and am interested to apply it myself in future work", and that "the method seems solid and the simulation results are promising". We thank the reviewers for their helpful comments which we feel have greatly improved our manuscript. In summary, we have made the following major modifications to the manuscript and method:

1. Stricter linkage disequilibrium (LD) clumping, and examination of how varying pairwise LD between signal clusters affects false positive (FP) rate in gene-to-trait slope estimation in simulation;
2. Examination of how varying instrument p-value cutoff affects FP rate in simulation;
3. New null simulations with gene-only and trait-only effects, in the absence of mediation, replacing previous null simulations;
4. New partial mediation simulations with spiked-in trait-only causal SNPs inducing horizontal pleiotropy through LD;
5. Comparison with eCAVIAR on colocalization step in simulation and real data;
6. Examination of relationship between low interval coverage and estimator bias;
7. Example of disagreement of MRLocus with other methods on a real data locus using eQTLGEN blood eQTLs.

We respond to each of the reviewers comments point by point below.

Reviewer 1:

> 1) The authors state in the introduction that MRLocus estimates gene-to-trait effects at loci containing ‘multiple LD-independent’ eQTL. However, in both their simulations and applied analyses they apply a threshold of r2 < 0.2 (based on the 1KG ref panel). I think this is slightly misleading to the reader, as in the applied analysis of SORT1 it suggests there are 12 ‘independent’ eQTL in liver tissue. However, based on this threshold I think that these 12 variants may well be weakly correlated with each other. In a conventional MR analysis I would be very skeptical of an analysis using this threshold as weakly correlated instruments are likely to result in ‘double counting’ which can bias findings.
>
> I therefore think it is important that the authors run simulations to investigate this, using a more stringent threshold i.e. r2<0.001 to compare how their method compares to other

approaches using truly independent instruments. Furthermore, I would like to see how false positive rates are affected when the r2 threshold is varied. My concern is that MRLocus may be prone to false positive findings using an r2 < 0.2, although it will be interesting to see how this compares to other methods in the field. Additionally, the instances of 'independent' eQTL should be changed if the authors can demonstrate that their method performs well under the default setting of r2<0.2.

We thank the reviewer for this suggestion, and we agree that clumping based on index SNPs using a $r^2 < 0.2$ threshold was too liberal in the initial submission, such that double counting likely would occur. We now use PLINK clumping with index SNP $r^2 < 0.1$ to identify nearly-LD-independent instruments, which is more commonly used in MR settings (see below). We further trim signal clusters prior to MRLocus input to maintain $r^2 < 0.05$ across pairs of clusters, before and after colocalization, to decrease the chances of double counting. Furthermore, we examine FP rate when varying signal cluster pairwise r^2 in a new simulation, where we find 0.05 to be a suitable cutoff for alleviating the effect of cluster r^2 on MRLocus slope estimation (Supplementary Figure 6). Finally, we have changed the language of the manuscript, replacing "LD-independent" with "nearly-LD-independent" to reflect that pairwise r^2 of up to 0.05 may, rarely, identify signal clusters driven by the same causal variant. We provide the pairwise r^2 of all signal clusters in all analyses (Supplementary Figure 7 for simulations, and Supplementary Figures 39-44 for real data analyses).

The $r^2 < 0.1$ clumping threshold has been used in previous Mendelian Randomization applications with QTLs. For example, Huang et al [1] retains cis-eQTL signal clusters that have $r^2 < 0.1$ as the instrumental variables in their MR analysis to examine the effect of gene expression on auto-immune mediated diseases. In the TWMR framework [2], the authors first run a stepwise procedure of conditional analysis to find all independent eQTLs [3] and then clumped the SNPs with $r^2 < 0.1$. Hannon et al [4] discussed using DNA-Methylation QTLs to characterize the causal relationship of methylomic variation on the gene expression and the complex traits. They used clumping with $r^2 < 0.1$ cutoff in PLINK as well when identifying independent association signals in a DNAm site before MR analysis.

Clumping r^2 references:

1. Huang, Qin Qin, Howard H. F. Tang, Shu Mei Teo, Danny Mok, Scott C. Ritchie, Artika P. Nath, Marta Brozynska, Agus Salim, Andrew Bakshi, Barbara J. Holt, Chiea Chuen Khor, Peter D. Sly, Patrick G. Holt, Kathryn E. Holt, and Michael Inouye. "Neonatal Genetics of Gene Expression Reveal Potential Origins of Autoimmune and Allergic Disease Risk." *Nature Communications* 11, no. 1 (2020). doi:10.1038/s41467-020-17477-x.
2. Porcu, Eleonora, Sina Rüeger, Kaido Lepik, Federico A. Santoni, Alexandre Reymond, and Zoltán Kutalik. "Mendelian Randomization Integrating GWAS and EQTL Data Reveals Genetic Determinants of Complex and Clinical Traits." *Nature Communications* 10, no. 1 (2019). doi:10.1038/s41467-019-10936-0.
3. Yang, Jian, Teresa Ferreira, Andrew P. Morris, Sarah E. Medland, Pamela A F Madden, Andrew C. Heath, Nicholas G. Martin, Grant W. Montgomery, Michael N. Weedon, Ruth J. Loos, Timothy M. Frayling, Mark I. Mccarthy, Joel N. Hirschhorn, Michael E. Goddard, and Peter M. Visscher. "Conditional and Joint Multiple-SNP Analysis of GWAS Summary

Statistics Identifies Additional Variants Influencing Complex Traits." *Nature Genetics* 44, no. 4 (2012): 369-75. doi:10.1038/ng.2213.

4. Hannon, Eilis, Tyler J. Gorrie-Stone, Melissa C. Smart, Joe Burrage, Amanda Hughes, Yanchun Bao, Meena Kumari, Leonard C. Schalkwyk, and Jonathan Mill. "Leveraging DNA-Methylation Quantitative-Trait Loci to Characterize the Relationship between Methylomic Variation, Gene Expression, and Complex Traits." The American Journal of Human Genetics. October 25, 2018.

> 2) Related to my previous point, I think it should be made clear in the applied analysis how many instruments are used at each locus. Although it is available to the reader in the supplementary material, making this clear in the main text means that the reader doesn't have to seek this information out. The authors highlight SORT1 which had n=12 instruments in their analysis, although for example MRAS only had a single eQTL instrument. Can the authors comment on whether their method provides added value when this is the case (i.e. single instrument MR) over say the eCAVIAR method which their method is built on?

We now include in the main text the number of instruments for the real data loci (third paragraph of Results section on real data loci), and we refer to Supplementary Figure 2 for the number of instruments in the simulated data. Using updated clumping thresholds, *SORT1* now has 6 instruments and *MRAS* has 9 instruments.

We note that previously, Supplementary Table 1 gave the "paired" eQTL and GWAS signal clusters, while additional "unpaired" signal clusters were included as well in slope fitting to ensure accurate estimation (all potential instruments included regardless of their effect size on the trait). Previously, we used a slightly different clumping approach on the real datasets as on the simulated datasets, which involved a more manual clumping strategy for signal clusters with evidence of colocalization. However, in the revised manuscript we use and recommend the same strategy in simulation and real datasets, so there are not "paired" vs "unpaired" signal clusters for the real data. All signal clusters are produced by clumping with PLINK followed by colocalization and trimming.

Additionally, we now state in the text and in the software guide (and have added software warnings) that MRLocus should only be used for loci with evidence of more than one nearly-LD-independent instrument.

> 3) I'm also concerned by the P<0.001 threshold applied to identify eQTL as instruments. Can the authors please evaluate how a more stringent threshold influences the false positive rates of their method compared to those it is benchmarked against? In a conventional MR setting an F-stat < 10 is typically used to indicate weak instruments, which will likely be the case when eQTL have P=10-03. This issue is not highlighted in this work as the authors have selected 5 genes which have strong prior knowledge that they are involved in disease risk/trait variation. However, if readers are interested in applying MRLocus to detect novel genes responsible for GWAS signals (which should

be its primary purpose I would've thought?) then the choice of p-value threshold will likely be important in mitigating false positive rates.

In my opinion applying conventional MR instrument criteria of P<5x10-08 and r2<0.001 is not necessary for cis-QTL analyses, however I think the authors should simulate this to investigate how these parameters influence the performance of their method. Some discussion regarding this would also be welcome for readers interested in applying their approach but perhaps have not studied MR extensively.

We note that clumping at P < 0.001 (corresponding to absolute Z-score of > ~3.3) provides index eSNPs as candidate instruments with F-statistic > ~10.8, so excluding what are often described as weak instruments (e.g. F-statistic < 10) in literature.

In order to determine if further thresholding would improve estimation or interval coverage, we now also consider clumping at P < 0.0001 (labelled with "_p1e-4" in Supplementary Figure 30, and corresponding to F-statistic > ~15.1), and include an additional simulation with higher eQTL sample size (raising from N=500 to N=1,000). We find that the methods that we benchmarked making use of PLINK clump (TWMR and MRLocus) perform mostly similarly, with slightly worse accuracy for MRLocus when restricting to F-statistic > ~15.1 for both N=500 and N=1,000 datasets, and slightly better accuracy for TWMR. However, the changes are slight, for example TWMR RMAE goes from 0.342 to 0.332, and MRLocus RMAE goes from 0.193 to 0.205 in simulation A (Supplementary Figure 30). Clumping at p-value < 0.0001 / F-statistic > ~15.1 does not improve interval coverage for MRLocus, but does slightly improve interval coverage for TWMR with coverage changing from 14% to 23% in simulation A for example (Supplementary Figure 30).

Overall MRLocus has better accuracy and coverage regardless of the clumping p-value threshold (p < 0.001 or p < 0.0001) compared to TWMR or PTWAS in the simulations. Our recommendation therefore based on Supplementary Figures 6 and 30 for MRLocus is to use PLINK clump with `--clump-p1 0.001` and `--clump-r2 0.1` and then trim clusters to obtain $r^2 < 0.05$ across instruments provided to MRLocus slope estimation.

4) Although the authors have used the 1KG reference panel as their study for LD calculations and clumping, this dataset consisting of 503 European individuals which based on modern standards is severely outdated. Although I won't ask the authors to re-run their analyses, I would ask that they mention in their discussion that their method (as with others in the field) would really benefit from a larger reference panel for more accurate LD calculations. Even though they have restricted variants to MAF > 0.01, n=503 is going to be unreliable for low frequency SNPs in my experience.

We thank the reviewer for bringing up this critical limitation of the 1KG reference panel. We have added relevant text to the Discussion that larger reference panels would provide more accurate LD calculation and benefit these analyses. In addition, some of the authors of the manuscript are developing TopLD, a tool to explore LD based on TOPMed whole genome

sequencing data, (http://topld.genetics.unc.edu/topld/). We have added this also to the Discussion.

> 5) The authors do an excellent job of providing a summary of genetic colocalization & TWAS methodologies in their introduction, as well as why introducing MR to address the challenges of deciphering GWAS signals is important. This is a minor suggestion, but I felt that paragraphs 4-6 of the introduction slightly got away from the core message of this paper and may be slightly overwhelming for readers not primarily involved in this topic of research. Perhaps these paragraphs could therefore be streamlined, focusing on the methods which are used to benchmark MRLocus, rather than the plethora of other approaches in this very busy area of research.

We have attempted to streamline the Introduction to focus more closely on MRLocus' core task and mention of its direct competitors. We have moved critical details regarding the methods used for comparison to MRLocus down to the Methods section "Choice of methods for comparison". Paragraphs 4 and 5 are now collapsed into one, introducing MR with gene expression as mediator, and paragraph 6 has been trimmed.

~~~~~~~~~~~~

Reviewer 2:

> Major comments:
>
> #1 It would be clearer if the authors stated the exact mathematical model (in equations) for the simulation process instead of referring to another publication and software (twas_sim). It is not clear for example how much horizontal pleiotropy was added to the signals (i.e. violation of the MR assumption)?

We have now added to the Methods section the mathematical model of the twas_sim simulation in equations and using more clear terminology (as discussed below).

We have modified the three null simulation settings to involve horizontal pleiotropy, wherein genetic variants in the same locus are associated with gene expression and GWAS trait, but the gene does not mediate any of the trait variance (0% expression mediated trait variance explained).

We have also modified the twas_sim simulation framework to allow simulation of a locus with a gene where expression level partially mediates the GWAS trait, and some amount of horizontal pleiotropy. The simulation of partial mediation with horizontal pleiotropy (referred to as "HP" in the manuscript) is accomplished by adding SNPs with large effects on the trait that are *not* mediated through the gene (see further details and example region in response to comment #6 below). This modified simulation code is provided in the `hp` branch of the GitHub fork at
~~~~~~~~~~~~

`mikelove/twas_sim`. We compare TWMR, PTWAS, MRLocus, LDA-MR-Egger, and PMR-Summary-Egger using these new non-null horizontal pleiotropy simulations.

> #2 I feel there is a slight confusion between two concepts: expression mediated heritability [A] (which is the fraction of trait heritability mediated by gene expression) vs mediated trait variance explained [B] (as far as I understand from the authors' definition: the squared standardized (SD/SD) causal effect). For me the relationship between them is A = h2-expression x B, do I understand well? Would be great if the terminology could be clarified in the paper.

We thank the reviewer for highlighting this point that required clarification of language in the manuscript. Indeed the squared standardized causal effect is the trait variance explained by the mediator (e.g. assayed gene expression; called "B" by the reviewer), and the following relationship holds when expression and trait are standardized (as they are in the twas_sim simulations):

Expression mediated heritability (now *h2med*; called "A" by the reviewer) = h2 of gene-expression (*h2g*) x trait variance explained by gene expression (*alpha*^2; called "B" by the reviewer).

While the twas_sim software uses the argument "var-explained" for the expression mediated heritability, we agree that this is confusing and have replaced this term in the manuscript with "*h2med*". We have clarified this relationship in the Methods section.

Finally, we note that in the simulations we have the following relationship between parameters:

h2med ≅ h2g x alpha^2,

for example in the main simulation (twas_sim at default settings; Fig 2) 0.01 ≅ 0.1 x 0.32^2 (0.32 being the average true value for the parameter alpha [x-axis]), and the approximate relationship holds throughout all simulation settings.

We note that the value for true slope parameter alpha (x-axis in the simulation assessment plots; e.g. Fig 2A) varies across iterations in the simulation for fixed h2med and h2g. The reason for this variation is that the alpha provided by the twas_sim simulation framework corresponds to the gene-to-trait mediated effect, when eQTL and GWAS effect sizes are standardized using the SD of gene expression and trait in the samples (as would occur in real data analysis). The h2g and h2med in the twas_sim simulator describe the *population-level* expression heritability and the mediated trait heritability, respectively. As the eQTL and GWAS sample sizes grow (e.g. already seen in the eQTL N=1,000 simulation), the variation is reduced and we have alpha values converging to sqrt(h2med/h2g). This note is also provided in the Methods section on the simulation, and in the Supplementary Methods, section 1.2.3 on fitting the slope alpha.

> #3 While I see the difference between the colocalization step of MRLocus and eCAVIAR, I'm not sure how different results they would give. (Z-scores can be easily transformed back to effect sizes.) Have the authors compared head-to-head their approach to eCAVIAR when applied to the simulated data set to show superior performance?

We have now run eCAVIAR as an alternative to MRLocus for colocalization (step 1), and ahead of MRLocus gene-to-trait slope fitting (step 2). We find that eCAVIAR does outperform MRLocus on the non-null simulations in its ability to detect the true causal SNP (for all 11 non-null simulations, Supplementary Figure 31), though we note that eCAVIAR does not provide posterior estimates of effect size which were the originally intended input to MRLocus slope fitting step. When providing eCAVIAR-chosen SNPs and their original estimated coefficients from eQTL and GWAS to MRLocus slope estimation ("eCAVIAR-MRLocus"), gene-to-trait effect estimates were more accurate for 2 out of 11 non-null simulations (simulation A and H, Fig 2A and Supplementary Figure 18) compared to slope estimation using MRLocus-chosen SNPs and MRLocus posterior estimates of eQTL and GWAS effect size ("MRLocus"). This comparison is included in the Results and in the Discussion.

However, for the null simulations, in which eQTL eSNPs and GWAS trait-associated SNPs are distinct and expression does not mediate trait variance, MRLocus-chosen SNPs are always more accurate at detecting the true causal eSNP (e.g. the instruments for the MR step, Supplementary Figure 31). We believe this is because MRLocus is uniquely positioned to find the largest posterior effect sizes for eQTL which are used for SNP selection for slope fitting. MRLocus-chosen SNPs are also better at providing intervals that cover the truth (alpha=0) in the null simulations (Supplementary Figure 29).

We find that the combined method, eCAVIAR-MRLocus, overall is a suitable alternative to MRLocus for both colocalization and slope fitting, and have incorporated recommended steps into the Methods, our interpretation of the advantages and disadvantage of either approach into the Discussion, and the optional use of eCAVIAR for colocalization into the MRLocus software guide.

> #4 "For all simulations, if there were no SNPs in the simulated locus with eQTL un-adjusted p-value < 0.001 then a new seed was drawn." – This leads to Winner's curse bias, since you only use summary statistics that (by chance) pass the significance threshold, i.e. their effects will be overestimated and hence lead to an underestimation of the causal effect.

We now discuss this as a limitation in the Results, that effects of eQTLs in the simulations could exhibit over-estimation of eQTL effect sizes due to choice of loci that have at least one eQTL un-adjusted p-value < 0.001. Well-powered eQTL studies will suffer less from the Winner's Curse bias; in our simulations we used N = 500 and N = 1,000. We therefore examine the over-estimation of coefficients for the index eSNP across all simulations, and find that the distribution of the ratio of (estimate/true value) to be roughly centered on 1, with histograms showing both over- and under-estimation (Supplementary Figure 4). Over-estimation, and

mis-estimation in general, is reduced as gene expression heritability increases, as expected. We provide the median and IQR of the estimate/true ratios in the caption of Supplementary Figure 4. We recommend that loci with only weak instruments (all eQTL un-adjusted p-value > 0.001) not be used for mediation analysis.

> #5 Low coverage can be a consequence of two issues: (unaccounted) bias and/or badly calibrated SE. It would be helpful if the authors could explore what is the main reason for low coverage of all methods. To me it stems mostly from bias – can the authors show how methods compare in terms of bias?

We now calculate the bias, as the mean of the estimates of alpha across simulation iterations minus the *population-level* alpha, using the equation sqrt(h2med/h2g). We find, as can be seen in the simulation evaluation figures, that TWMR and PTWAS tend to have more negative bias, and we have provided bias estimates for simulation A in the second paragraph of Results. However, even if we adjust method performance by an oracle bias estimate, we still do not obtain reliable target coverage for TWMR or PTWAS across all the simulations. Therefore, these methods are both biased and have some problem with calibration of SE. We have added this as Supplementary Figure 32 where we explore interval coverage after oracle bias correction for the three 10% h2g simulations (h2med = {0.01, 0.005, 0.001}). We provide the bias for all three 10% h2g simulations in the caption of Supplementary Figure 32.

> #6 Fig 2A shows that MRLocus outperforms the oracle (known causal SNPs) both in terms of RMAE/MAE. The authors argue that it is due to the re-estimation of the effects using colocalization.

The outperformance by MRLocus compared to the oracle method ("causal", using causal SNPs) in Fig 2A in terms of RMAE (this was the only simulation in which this occurred) was due to an small error in the scaling of the true slope parameter in the twas_sim codebase used in the first submission. The true slope was not properly scaled for the standardization of the eQTL effect sizes within the simulation framework. After correcting this error (now incorporated into the original twas_sim simulation GitHub repository), the oracle estimator always outperforms all methods without access to the true causal SNP, as would be expected. Generally though, ranking among methods did not change greatly after fixing this bug, as the scaling effect was moderate.

> #6 cont. This however would only work if there is no pleiotropy present at all. We know from real data, that there is almost always pleiotropy present, so simulation settings must be extended to more realistic situations. (E.g. TWMR has an outlier removal step, analogous to MR-PRESSO to protect against such situations) In addition: it seems to me that in the simulations there were no trait-only effects simulated, which is again a very important omission. Gene->trait causal effects do not mean that all trait-associated SNP at the locus must be expression associated, traits can have additional genetic components. Adding this more realistic setting would show the weakness of MRLocus, because it would artificially pull up the effect of those SNPs on expression. Both these

situations should be incorporated into the simulations, otherwise the settings are too artificial and unrealistic.

In order to demonstrate the use of MRLocus on loci exhibiting horizontal pleiotropy, we have 1) modified the null simulations to now include trait-only effects, replacing previous null simulations (Supplementary Figure 29), and 2) added non-null simulations for loci in which there are a combination of trait-only effects and mediated effects (see an example locus below which is Supplementary Figure 8, and Supplementary Figures 9-10). For the non-null simulations combining trait-only and mediated effects, for each of the main simulation (simulation A) iterations, we add three large trait-only causal SNPs, where the trait variance is *not* mediated by gene expression level. We refer to these non-null simulations as "HP" or "horizontal pleiotropy" in the manuscript. As expected, these simulations induce higher error in estimation of the gene-to-trait effect for all methods (even the oracle method worsens, as the GWAS estimated coefficients for expression-mediated causal SNPs are modified via LD with the trait-only associations). However, we find in these simulations that MRLocus still outperforms existing methods both in terms of error and interval coverage.

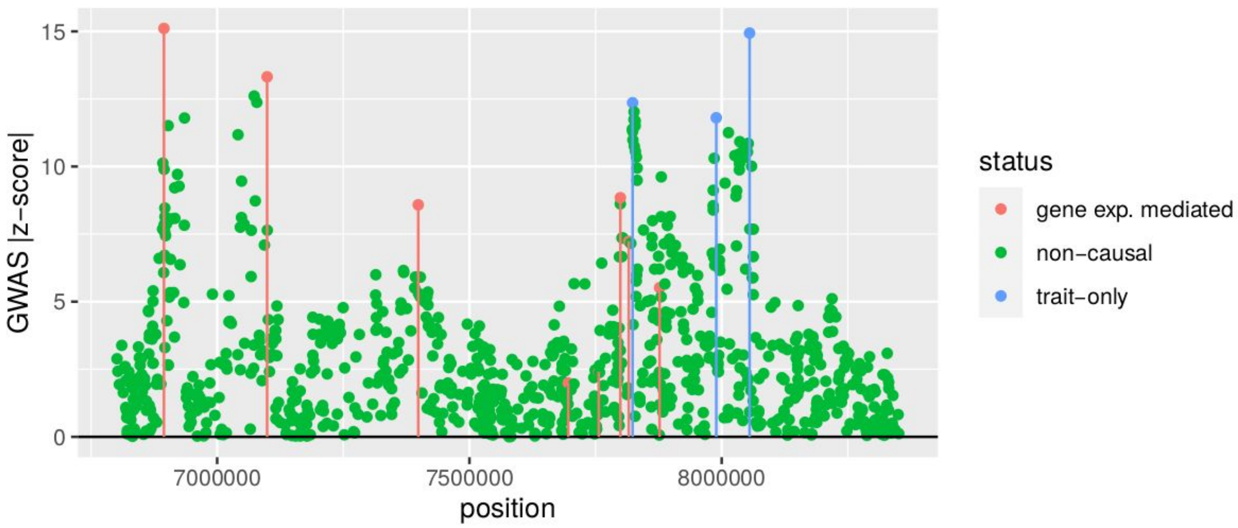

#7 While it is reassuring to see that previous findings are confirmed by MRLocus, but would be nice to see examples of gene-trait associations missed by other methods and only picked up by MRLocus or potentially spurious causal effects (by TWMR or PTWAS) disproved by MRLocus.

We have now added an example where we do not believe there is strong evidence of gene-to-trait mediation (blood eQTL using eQTLGEN summary statistics), but where other methods have strong evidence of causal effects based on confidence intervals. The negative association of LIPC with HDL in the liver has already been widely reported in literature. While we cannot rule out that blood tissue would have a strong *opposite* effect for the *LIPC* gene, the plot of the MR instruments (Supplementary Figures 45 [left plot included below] and 46) clearly

displays heterogeneity that is not well reflected in the confidence intervals of TWMR or PTWAS for this example (Fig 3, right plot included below).

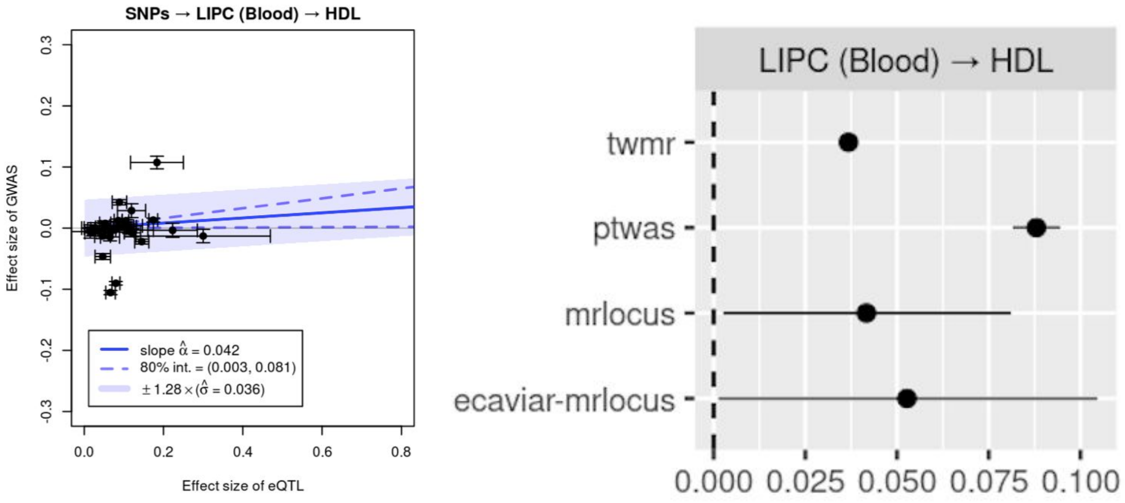

#8 Since the authors point out the importance of allelic heterogeneity, larger eQTL data sets, such as the eQTL-Gen summary statistics (whole blood, n=32K) would be more ideal to demonstrate the advantages of their method. Have the authors tried MRLocus on such data?

We have now added a simulation with larger eQTL sample size (N=1,000), where indeed all methods have higher accuracy at estimating the gene-to-trait effect. The higher accuracy is due to more nearly-LD-independent signal clusters recoverable by DAP, eCAVIAR and MRLocus (Supplementary Figure 11 and 30).

We also add a real data example using eQTLGen summary statistics: the *LIPC* gene in blood tested for mediation of HDL in a GWAS population. As described in Results, we feel the results of MRLocus are more plausible given the high heterogeneity of GWAS effect size for expression increasing alleles. However, we caution in the Discussion that the p-value threshold may need to be modified for such highly powered eQTL input data.

#9 I'd move quite some part of the supplementary methods (especially the method description) to the main text Methods – especially given that PLoS Gen has no strict page limit.

While we prefer to keep the full mathematical details of the method in the Supplementary Methods, we agree that there was insufficient description of the methods in the main text in the first submission. In the revision we have added the critical equations for the hierarchical model used in estimation of the gene-to-trait slope to the main text Methods.

> Minor comments:
>
> “the original TWMR paper performed conditional analyses” – TWMR performs approximate conditional analysis, which is designed for summary statistics.

We have now corrected the manuscript with respect to this point. We encountered difficulty with approximate conditional analysis and so we have used clumping for identification of signal clusters.

> “We therefore considered even lower heritability of trait on gene expression of 0.5% and 0.1%” – Please rephrase this sentence, it is currently not understandable, how a trait can have a heritability on expression?

We thank the reviewer for identifying this erroneous sentence, and have corrected the text to "We therefore considered even lower percent of gene-expression-mediated heritability of the GWAS trait, h^2med of 0.5% and 0.1%..."

> Fig 4B vertical whiskers are missing to indicate SE.

Fig 5B did contain vertical whiskers for GWAS SE, but they are small relative to the eQTL SE (horizontal).
